# Supplementary material for: MASTER-NAADP: a membrane permeable precursor of the Ca2+ mobilizing second messenger NAADP
Source: Nat Commun. 2024 Sep 13;15:8008. doi: 10.1038/s41467-024-52024-y (PMC11399135; doi:10.1038/s41467-024-52024-y)
Supplement: Supplementary file 1 — Supplementary Information [file 41467_2024_52024_MOESM1_ESM.pdf]

Supplementary Information

MASTER-NAADP: a membrane permeable precursor of the Ca^{2+} mobilizing second messenger NAADP

Sarah Krukenberg[1*], Franziska Möckl[2*], Mariella Weiß[2], Patrick Dekiert[1], Melanie Hofmann[1], Fynn Gerlach[2], Kai J. Winterberg[2], Dejan Kovacevic[2], Imrankhan Khansahib[2], Berit Troost[2], Macarena Hinrichs[2], Viviana Granato[2], Mikolaj Nawrocki[3], Tobis Hub [4,5], Volodymyr Tsvilovskyy[4,5], Rebekka Medert [4,5], Lena-Marie Woelk[6], Fritz Förster[6], Li Huan[7,8] , René Werner[6], Marcus Altfeld[9], Samuel Huber[3], Oliver Biggs Clarke[7,8], Marc Freichel[4,5], Björn-Philipp Diercks[2], Chris Meier[1#], Andreas H. Guse[2#§]

[1]Organic Chemistry, University of Hamburg, 20146 Hamburg, Germany

[2]The Calcium Signalling Group, Department of Biochemistry and Molecular Cell Biology, University Medical Center Hamburg-Eppendorf, 20246 Hamburg, Germany.

[3]Section of Molecular Immunology und Gastroenterology, I. Department of Medicine, University Medical Center Hamburg-Eppendorf, 20246 Hamburg, Germany.

[4]Institute of Pharmacology, Heidelberg University, Heidelberg, Germany

[5]DZHK (German Centre for Cardiovascular Research), partner site Heidelberg/Mannheim, Heidelberg, Germany

[6]Department of Applied Medical Informatics, University Medical Center Hamburg-Eppendorf, 20246 Hamburg, Germany.

[7]Department of Anesthesiology, Columbia University Irving Medical Center, New York, NY, USA.

[8]Department of Physiology and Cellular Biophysics, Columbia University, New York, NY, USA.

[9]Department of Immunology, University Medical Center Hamburg-Eppendorf, 20246 Hamburg, Germany.

[§]Corresponding author: Further information and requests for resources and reagents should be directed to and will be fulfilled by the lead contact, Andreas H. Guse (guse@uke.de)

Supplementary Figures

Supplementary Fig. 1: Synthesis of MASTER-NADP derivatives I (synthesis of the "northern" part) Reagents and conditions.

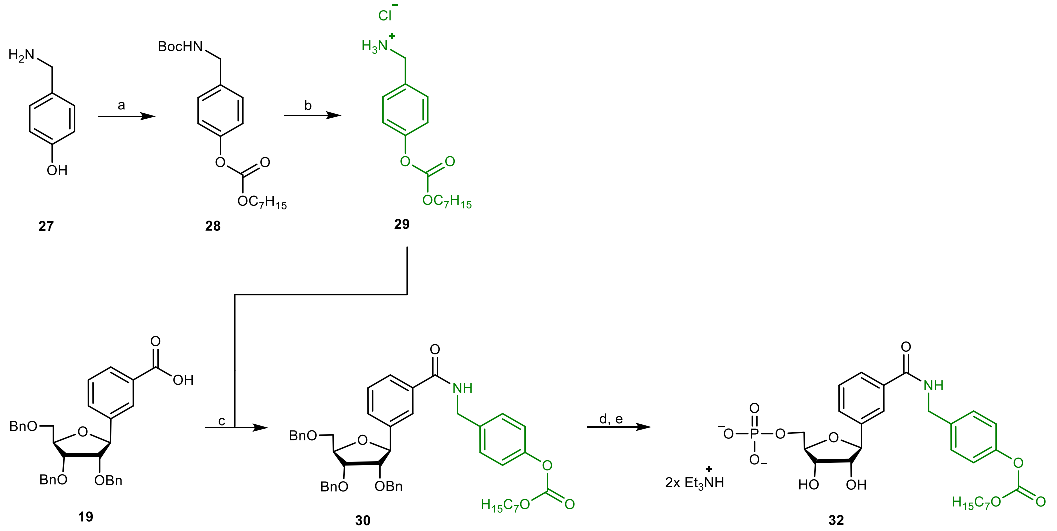

Reagents and conditions. [a] 1.1 eq. Boc_2O, DMF/pyridine (5:1), 3 h, rt, then 1.0 eq. Et_3N, 0.90 eq. **21**, CH_2Cl_2, 15 min, 0 °C, then 3 h, rt, 57 %; **[b]** 20 eq. AcCl, EtOH, 3 h, 0 °C → rt, 90 %. **[c]** 1.2 eq. HBTU, 2.2 eq. Et_3N, 1.1 eq. **29**, 0.3 eq. DMAP, CH_2Cl_2, 16 h, rt, 24 %; **[d]** 5.3 eq. BCl_3 (1 M, CH_2Cl_2), 45 min, -78 °C → -10 °C 83 %; **[e]** 2.0 eq. $P(O)Cl_3$, 2.5 eq. nBu_3N, trimethylphosphate, 16 h, 0 °C, quantitatively.

Supplementary Fig. 2: Synthesis of MASTER-NADP derivatives II (synthesis of the "southern" part and coupling with the "northern" part)

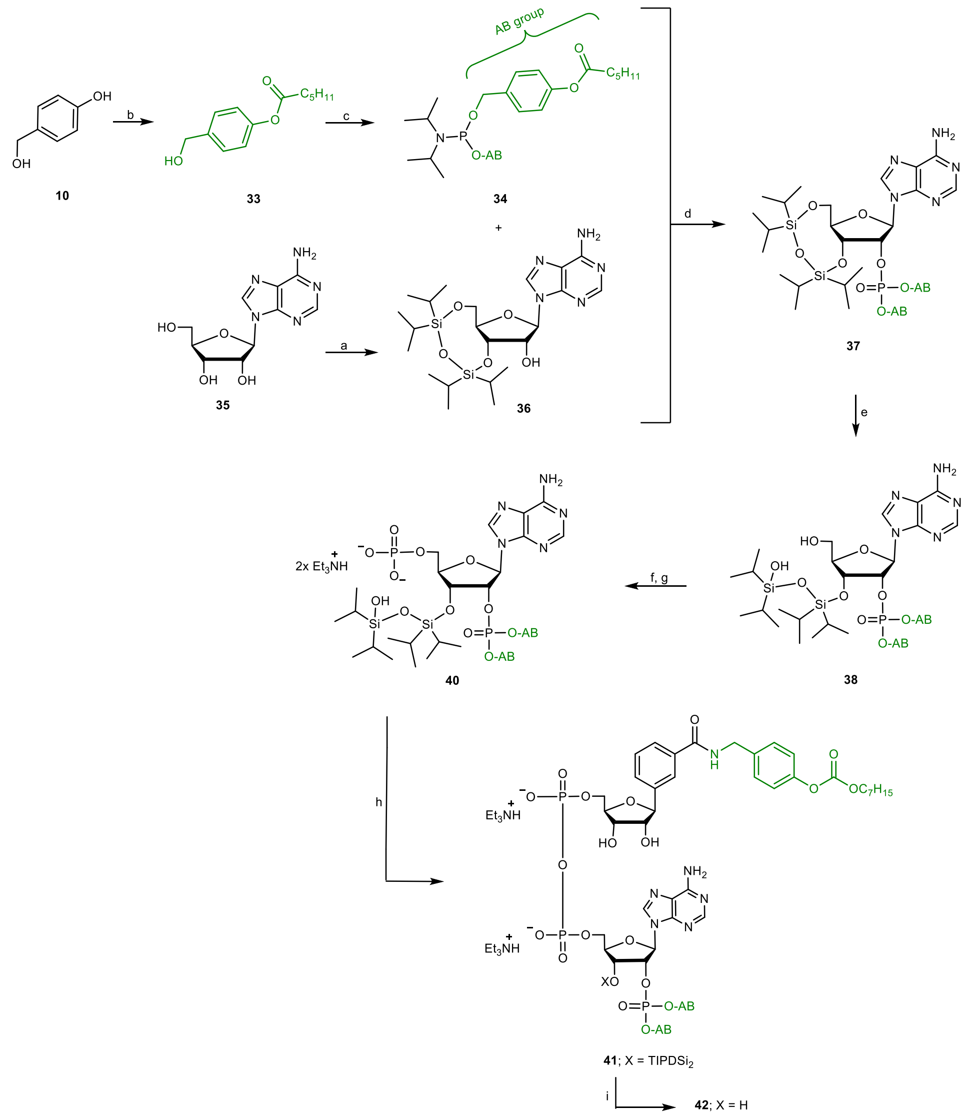

Reagents and conditions. **[a]** 1.1 eq. $TIPDSiCl_2$, 0.5 eq. DMAP, pyridine, 16 h, rt, 91 %; **[b]** 1.1 eq. 4-hydroxyl benzyl alcohol **10**, 1.0 eq. Et_3N, 1.0 eq. hexanoyl chloride, CH_2Cl_2, 0 °C, 3 h, 44 %; **[c]** 1.0 eq. dichloro-*N,N*-di*iso*-propylaminophosphoramidite, 2.0 eq. **33**, 2.2 eq. Et_3N, THF, -20 °C → rt, 16 h, 72 %; **[d]** 1.1 eq. **34**, 1.5 eq. DCI 0.25 M, CH_2Cl_2, 2 h, 0 °C → rt, 2.0 eq. *tert*.-BuOOH, CH_2Cl_2, 1 h, rt, 70 %; **[e]** THF:H_2O:TFA, 3 h, 0 °C, 83 %; **[f]** 5.0 eq. imidazole, 3.5 eq. Et_3N, 1.1 eq. PCl_3, CH_2Cl_2, 15 min, -5 °C, TEAB-buffer 1 M, 15 min, rt, 89 %; **[g]** 5.0 eq. BSA, CH_2Cl_2, 1 h, rt, 3.0 eq. CSO, CH_2Cl_2, 1 h, rt, TEAB-buffer (1 M)/ CH_3OH (1:1), 15 min, rt, 99 %; **[h]** "northern" part **32**, 10.0 eq. TFAA, 16.0 eq. Et_3N, CH_3CN, 10 min, 0 °C → rt, then 6.0 eq. NMI, 10.0 eq. Et_3N, CH_3CN, 10 min, 0 °C → rt, 1.1 eq. **40**, CH_3CN, 3 h, rt, then TEAB-buffer 1 M, 15 min, rt, 64 %; **[i]** 2.0 eq. TASF, 4.0 eq. H_2O, CH_3CN, 1 h, 0 °C, 55 %.

Supplementary Fig. 3: HPLC analysis of purity and digestion of MASTER-NADP by porcine liver esterases

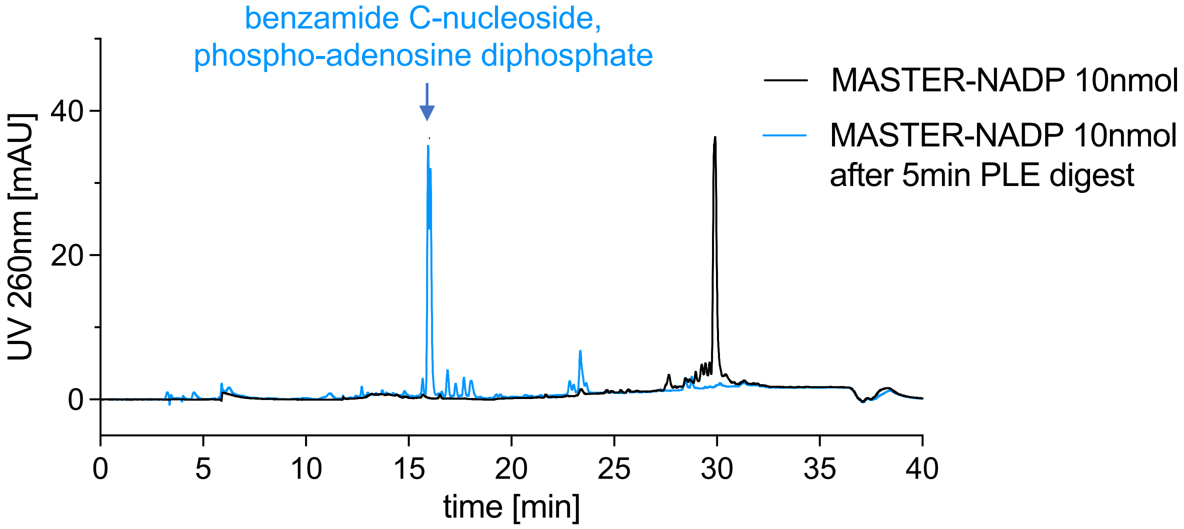

MASTER-NADP was first analyzed for purity by RP-HPLC as described in Materials and Methods. In short, for separation of components a 250 mm x 4.6 mm C8 Luna column (5 µm particle size, Phenomenex) was used as stationary phase. Further, HPLC buffer A (20 mM KH_2PO_4, pH 6.0) and B (10% buffer A, 90% methanol) served as the mobile phase. By increasing the methanol content in the mobile phase nucleotides were eluted from the column. Nucleotides were detected at 260nm using a Diode-Array detector (DAD, Agilent Technologies). MASTER-NADP was then digested with 5 U/ml porcine liver esterases (PLE; blue) for 5min at 37°C. HPLC chromatograms show MASTER-NADP before digestion (black), and its main digestion products, the 2'- and 3'-phosphate isomers of benzamide C-nucleoside, phospho-adenosine-diphosphate (blue). A representative out of 4 experiments is shown. Source data are provided as a Source Data file.

Supplementary Fig. 4: MASTER-NAADP does not evoke local Ca^{2+} microdomains in *Hn1l/Jpt2*$^{-/-}$ cells

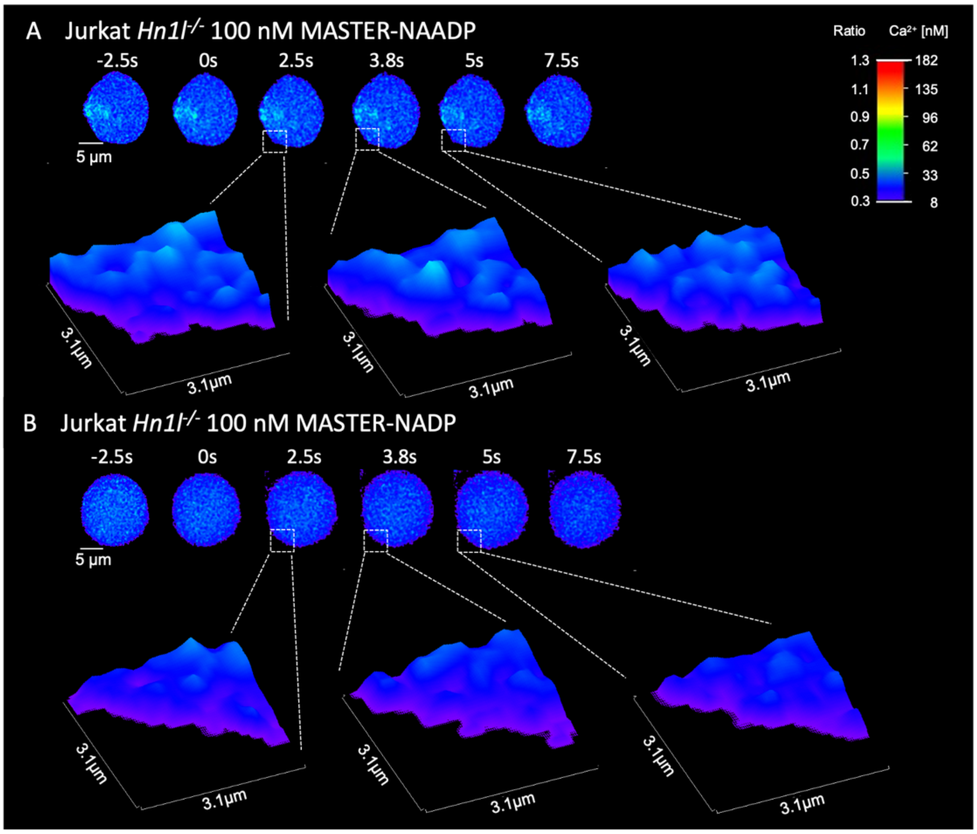

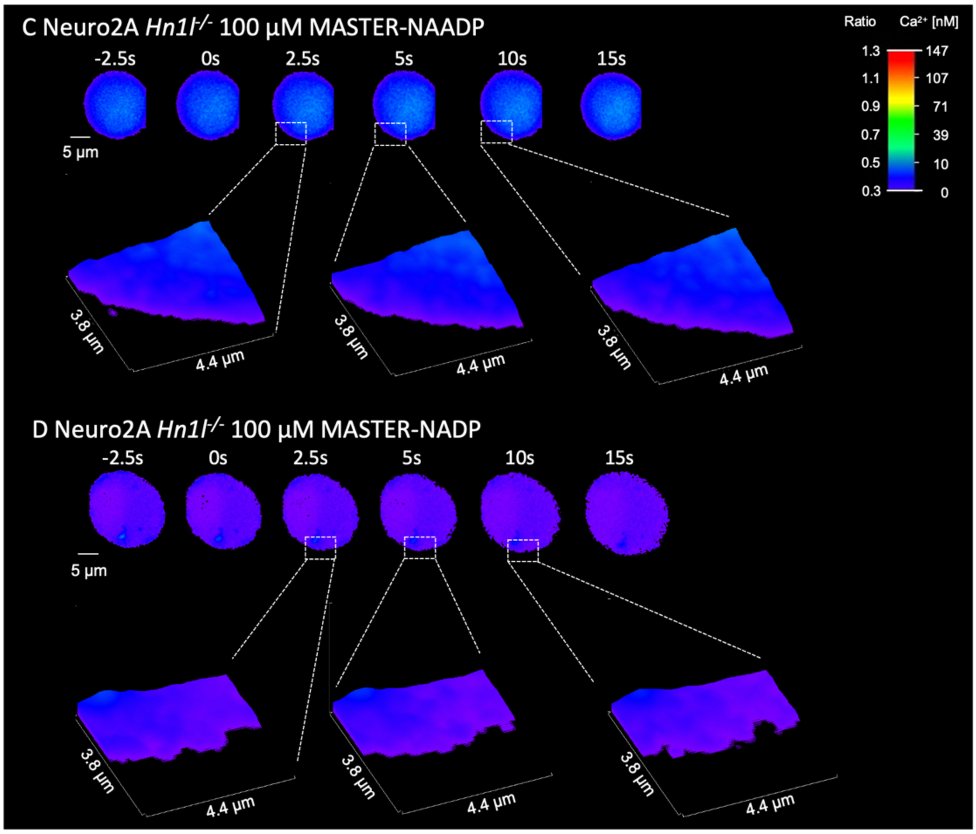

Representative high-resolution Ca^{2+} images of Jurkat *Hn1l/Jpt2*$^{-/-}$ T cells (**A**, **B**) and Neuro2A *Hn1l/Jpt2*$^{-/-}$ cells (**C**, **D**) loaded with both Fluo4-AM and Fura-Red-AM after stimulation with MASTER-NAADP (**A**, **C**) or MASTER-NADP (**B**, **D**). In Jurkat *Hn1l/Jpt2*$^{-/-}$ T cells 100nM was used, whereas 100µM MASTER-compound was applied in Neuro2A *Hn1l/Jpt2*$^{-/-}$ cells. For Jurkat *Hn1l/Jpt2*$^{-/-}$ T cells (**A**, **B**) the heatmap indicates emission ratios between Fluo-4 and Fura-Red ranging from 0.3 -1.3; ratio data were then converted using external calibration corresponding to 8 to 182 nM $[Ca^{2+}]_i$. Magnified regions as indicated as 3D surface plots. Jurkat *Hn1l/Jpt2*$^{-/-}$ MASTER-NAADP, n = 43 cells; Jurkat *Hn1l/Jpt2*$^{-/-}$ MASTER-NADP, n = 37 cells. For Neuro2A *Hn1l/Jpt2*$^{-/-}$ cells (**C**, **D**) the heatmap indicates emission ratios between Fluo-4 and Fura-Red ranging from 0.3 – 1.3; ratio data were then converted using external calibration corresponding to 0 to 147 nM $[Ca^{2+}]_i$. Scale bars, 5 µm for whole cells. Neuro2A *Hn1l/Jpt2*$^{-/-}$ MASTER-NAADP, n = 21 cells; Neuro2A *Hn1l/Jpt2*$^{-/-}$ MASTER-NADP, n = 20 cells

Supplementary Fig. 5: Dartboard projection of Ca^{2+} microdomains of shape normalized Jurkat WT and *Hn1l/Jpt2$^{-/-}$* T cells.

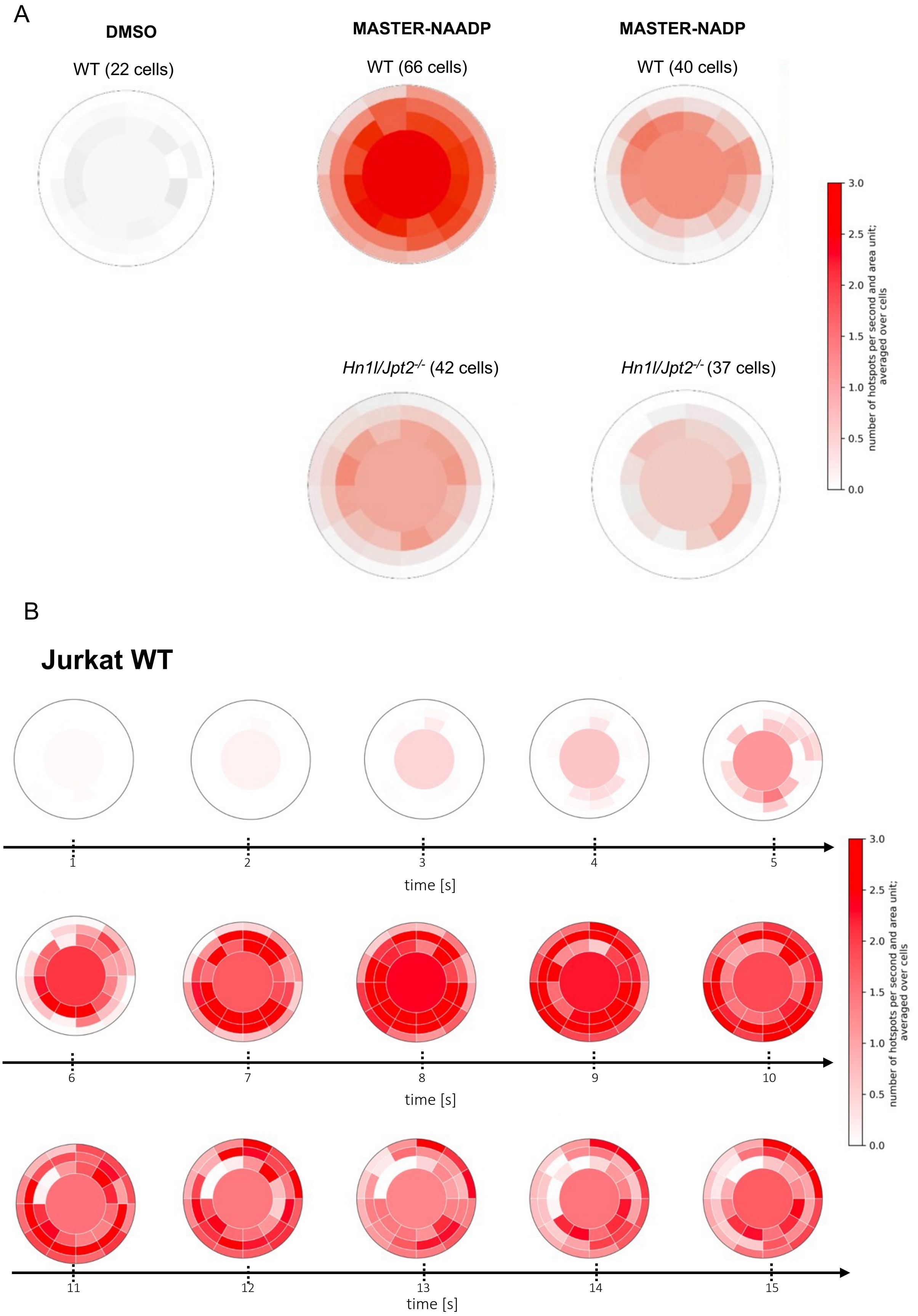

(A) Dartboard projections of shape normalized Jurkat WT and *Hn1l/Jpt2*$^{-/-}$ cells stimulated with 100 nM of MASTER-NAADP or MASTER NADP, or DMSO control. Depicted are the aggregated mean Ca^{2+} microdomains of the cell populations from Fig 4C over the initial 15 seconds after activation. The color bar indicates a maximum value of 3 Ca^{2+} microdomains per segment within 15s. **(B)** Timeline of subtracted dartboard projection plots of shape normalized Jurkat WT cells in second steps (data from Fig 4C). Here, for every second after stimulation, the aggregated mean Ca^{2+} microdomains from WT Jurkat cells stimulated with MASTER-NADP (control compound) were subtracted from MASTER-NAADP of each dartboard segment. Hence, the formation and localization of Ca^{2+} microdomains upon MASTER-NAADP addition are visualized inside the Jurkat WT cells over the first 15s in 1s steps. The color bar indicates a maximum value of 3 Ca^{2+} microdomains per segment within 1s.

Supplementary Fig. 6: Extended temporal analysis of MASTER-NAADP evoked Ca^{2+} microdomains in T cells

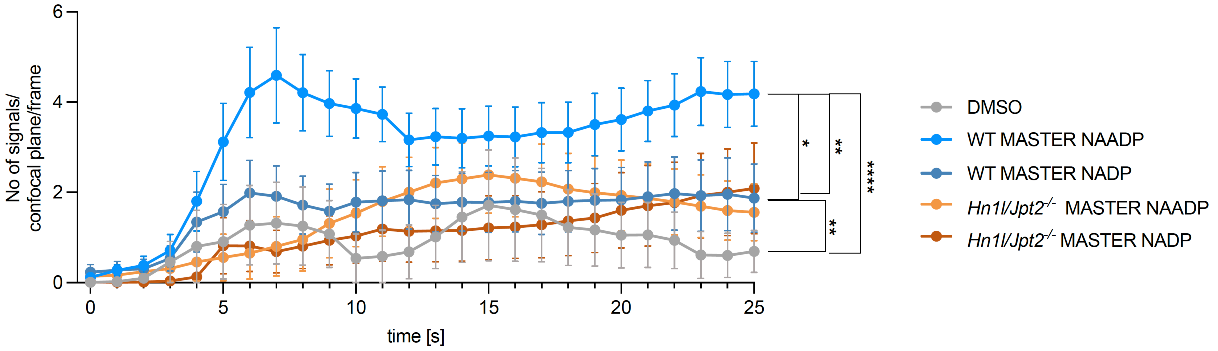

Extended analysis of the data shown in Fig 4C as a time course in 1s-steps. Analysis from 0 up to 25s after stimulation of Jurkat T cells shown as number of Ca^{2+} microdomains per confocal plane and frame. Data are displayed as mean ± SEM; WT MASTER-NAADP, n = 43 cells; WT MASTER-NADP, n = 37 cells; *Hn1l/Jpt2*$^{-/-}$ MASTER-NAADP, n = 66 cells; *Hn1l/Jpt2*$^{-/-}$ MASTER-NADP, n = 40 cells; WT DMSO, n = 17 cells; nonparametric Kruskal-Wallis test and Dunn's correction for multiple testing *P < 0.05; **P < 0.01; ****P < 0.0001. Source data and exact *p* values are provided as a Source Data file.

Supplementary Fig. 7: Dartboard projection of Ca^{2+} microdomains of shape normalized KHYG-1 WT cells.

A

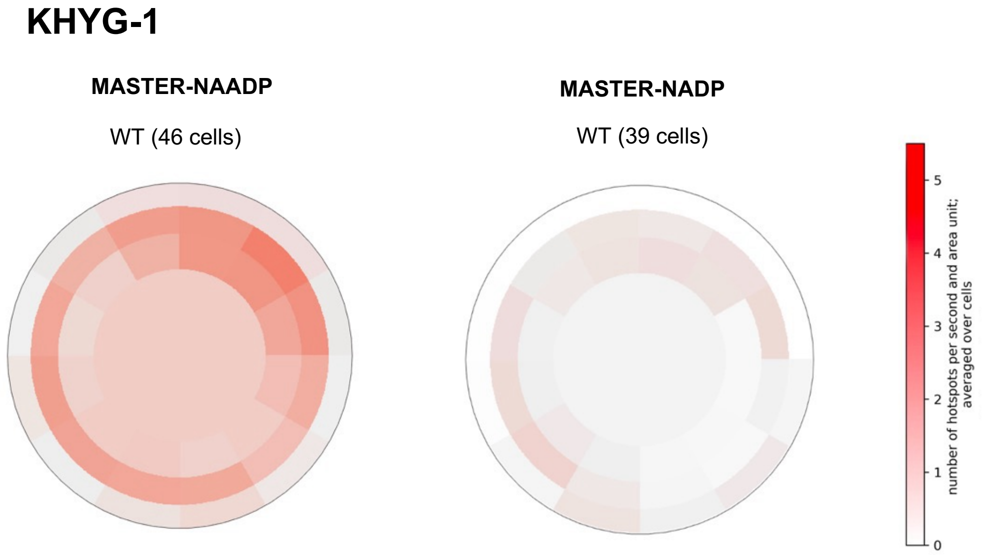

B

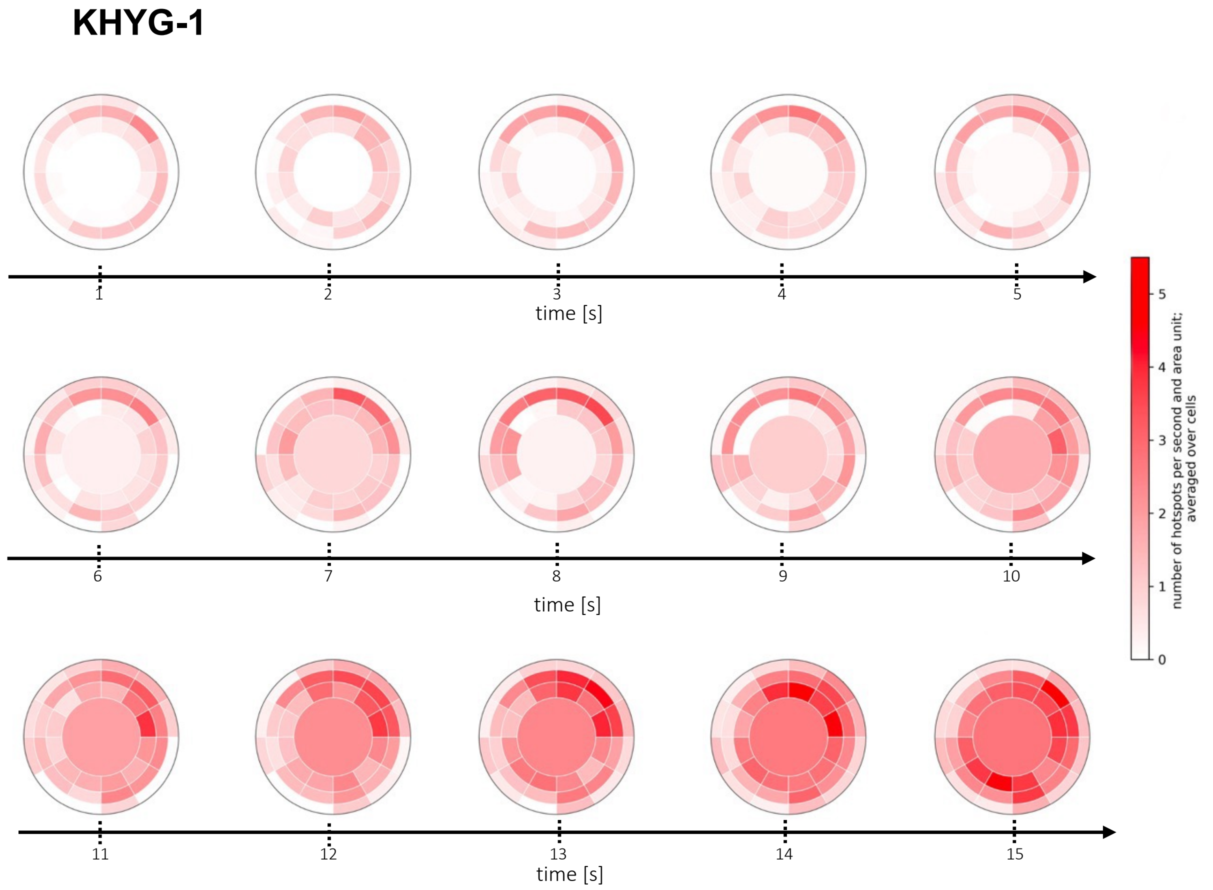

(**A**) Dartboard projections of shape normalized KHYG-1 cells stimulated with 100 µM of MASTER-NAADP or MASTER-NADP. Depicted are aggregated mean Ca^{2+} microdomains of the cell populations from Fig 4G over the initial 15 seconds after activation. The color bar indicates a maximum value of 5.5 Ca^{2}+ microdomains per segment within 15s. (**B**) Timeline of subtracted dartboard projection plots of

shape normalized KHYG-1 WT cells in one second steps (data from Fig 4G). Here, for every second after stimulation, the aggregated mean Ca^{2+} microdomains from KHYG-1 cells stimulated with MASTER-NADP (control compound) were subtracted from MASTER-NAADP of each dartboard segement. Hence, the formation and localization of Ca^{2+} microdomains upon MASTER-NAADP addition are visualized inside the KHYG-1 cells over the first 15s in 1s steps. Color bar indicates a maximum value of 5.5 Ca^{2+} microdomains per segment within 1s.

Supplementary Fig. 8: Comparison of Ca^{2+} signaling upon stimulation with MASTER-NAADP, MASTER-NADP, or NAADP-AM in Jurkat T cells

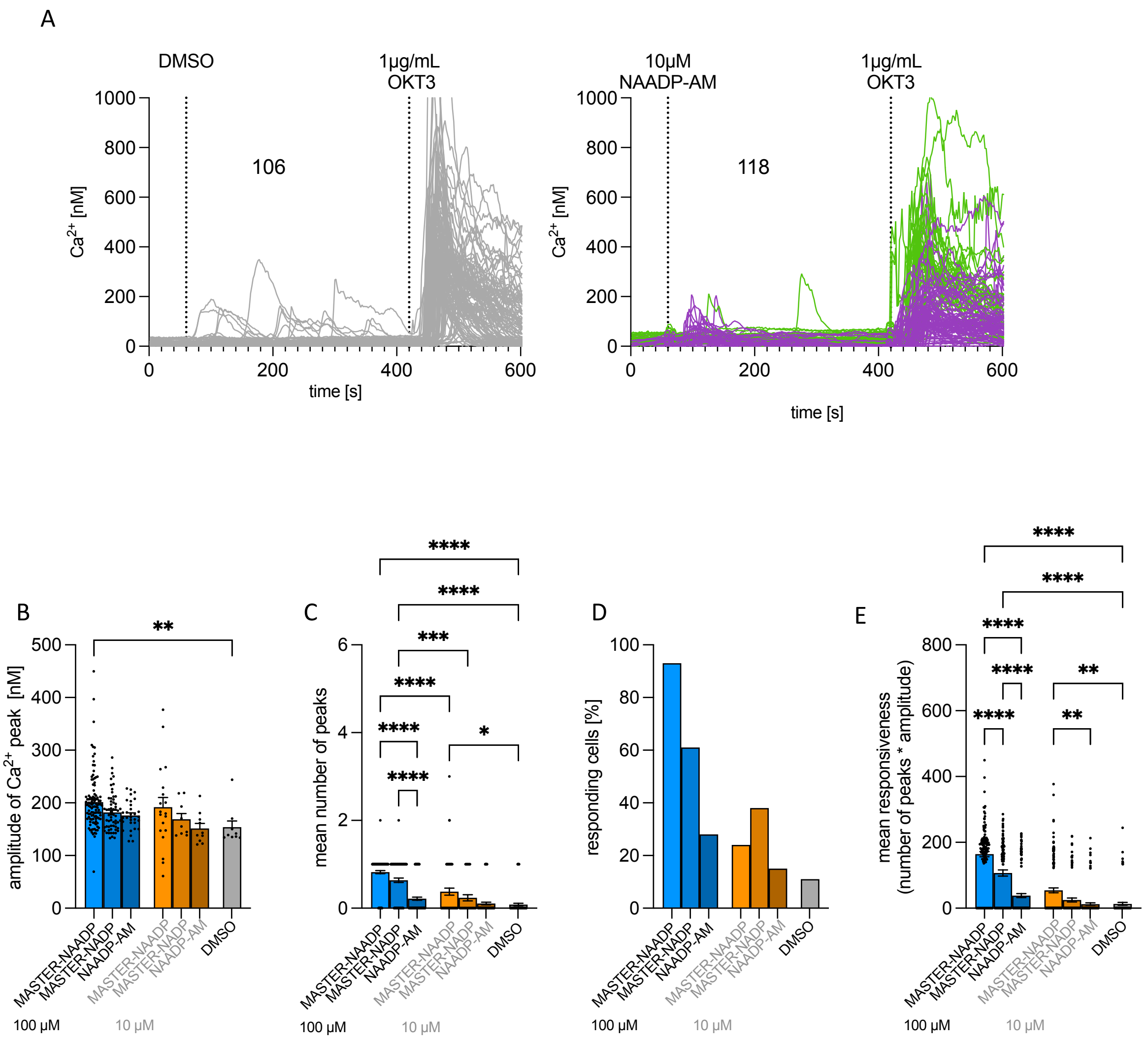

(**A**) Global Ca^{2+} signaling was analyzed in Fura2-loaded Jurkat WT T cells, upon addition of DMSO as vehicle control or 10µM NAADP-AM (green: lot #280181, purple: lot #3030795). To control for responsiveness of cells, anti-CD3 mAB OKT3 (1 µg/ml) was added at 420s. Experiments were carried out at 37°C and SOCE was blocked by pre-incubation of 50 µM Synta66 for 5 min prior to imaging. Aggregated data upon addition of 10µM or 100µM MASTER-NAADP, MASTER-NADP or NAADP-AM or DMSO as vehicle control as mean peak amplitude (**B**), mean number of Ca^{2+} peaks (**C**), percentage of responding cells (**D**), and calculation of the mean responsiveness (number of peaks * amplitude; **E**) presented as mean ± SEM; 100µM MASTER-NAADP, n = 134 cells; 10µM MASTER-NAADP, n = 61 cells; 100µM MASTER-NADP, n = 100 cells; 10µM MASTER-NADP, n = 38 cells; 100µM NAADP-AM, n = 143 cells; 10µM NAADP-AM, n = 120 cells; DMSO, n= 106 cells. Nonparametric Kruskal-Wallis test and Dunn's correction for multiple testing *P < 0.05; **P < 0.01; ***P < 0.001; ****P < 0.0001. Source data and exact *p* values are provided as a Source Data file.

Supplementary Fig. 9: Purity analysis of commercially available NAADP-AM

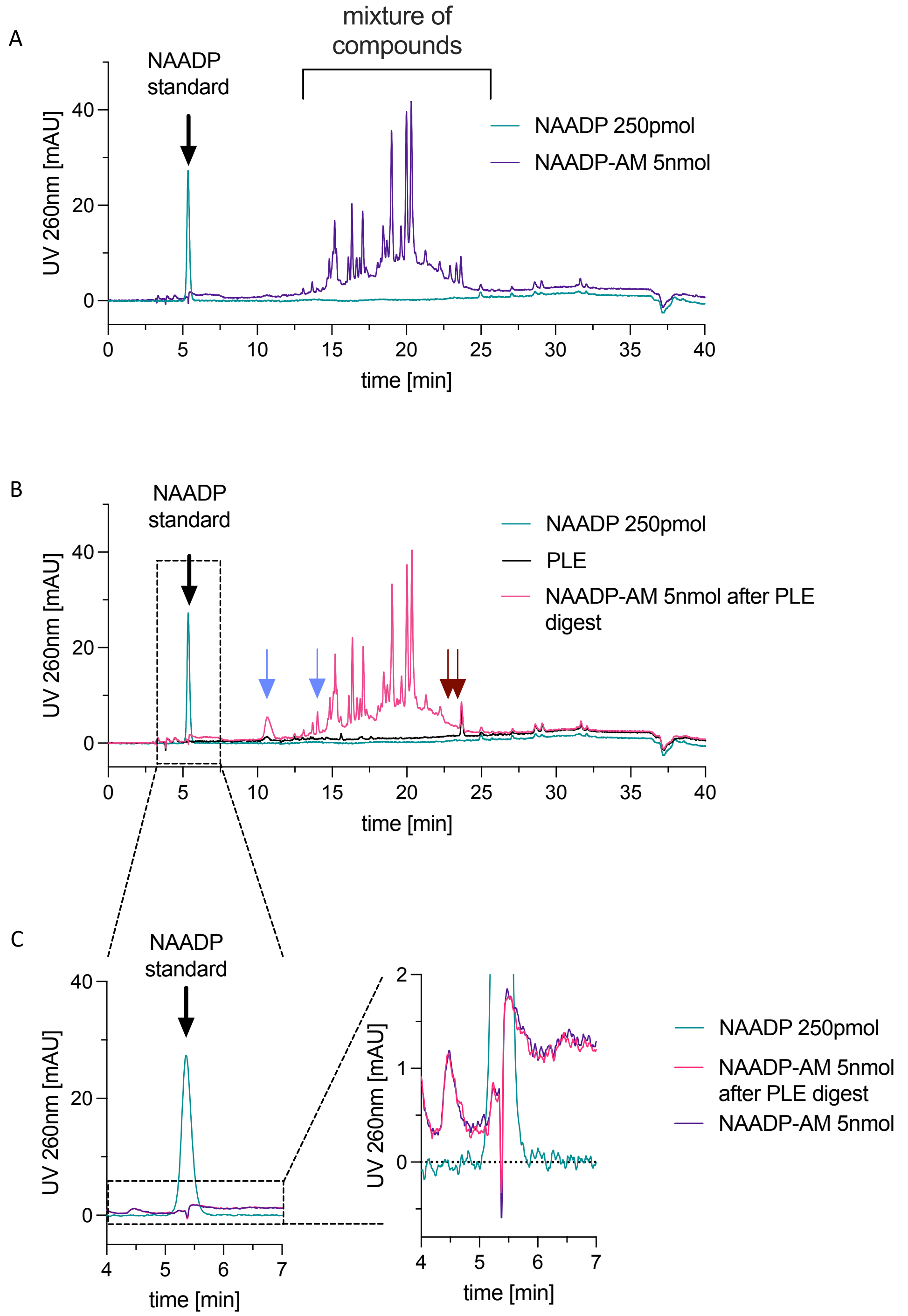

(A) HPLC analysis of commercially available NAADP-AM (5nmol) and NAADP (250 pmol) as control. **(B)** HPLC analysis of NAADP-AM (5nmol) after digestion with porcine liver esterase (PLE; pink) and PLE alone (black) and and NAADP (250 pmol; turquoise) as control. (C, left) Magnification of the the dashed area shown in **(B)** adding 5nmol un-digested NAADP-AM (violet). (**C**, right) further magnification of the dashed area shown in (**C**, left). A representative of 3 experiments is shown. Source data are provided as a Source Data file.

Supplementary Fig. 10: Purity analysis of four different lots of commercially available NAADP-AM

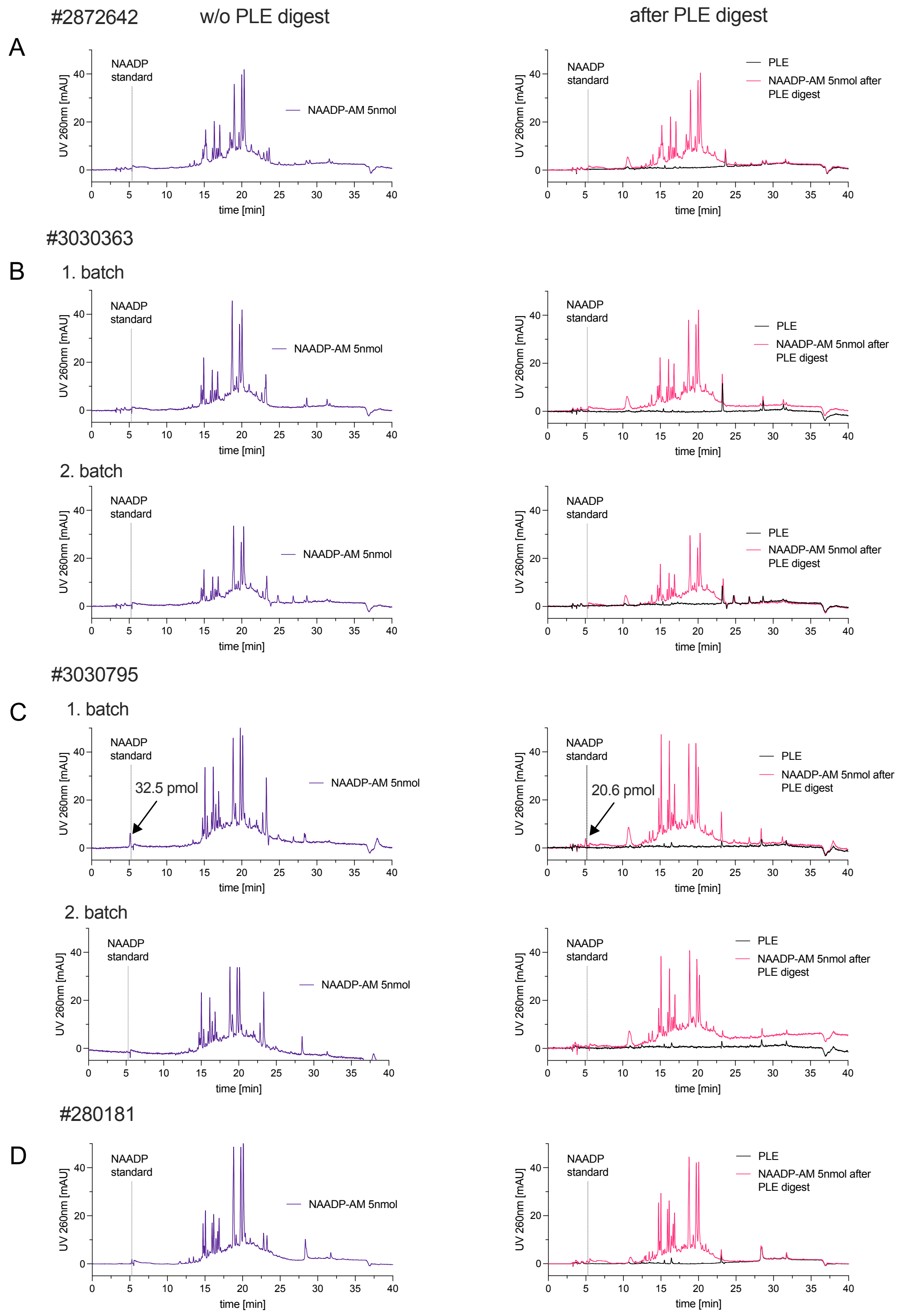

HPLC analysis of commercially available NAADP-AM (5nmol) (left panels) before (violet) and (right panels) after digestion with porcine liver esterase (PLE; pink) and PLE alone (black) for four different lots: (**A**) lot number #2872642, (**B**) lot number #3030363 (two individual batches are displayed), (**C**) lot number #3030363 (two individual batches are displayed) and (**D**) lot number #280181. The NAADP standard is indicated by a dashed line. NAADP can be detected in (**C**, batch 1) and was quantified as (left panel)

32.5 pmol before compared to (right panel) 20.6 pmol after digestion with PLE.Compounds were separated by reversed phase-HPLC as described in Materials and Methods. A representative of 3 experiments for each lot is shown. Source data are provided as a Source Data file.

Supplementary Fig. 11: Liberation of cAMP from cAMP-AM by porcine liver esterases

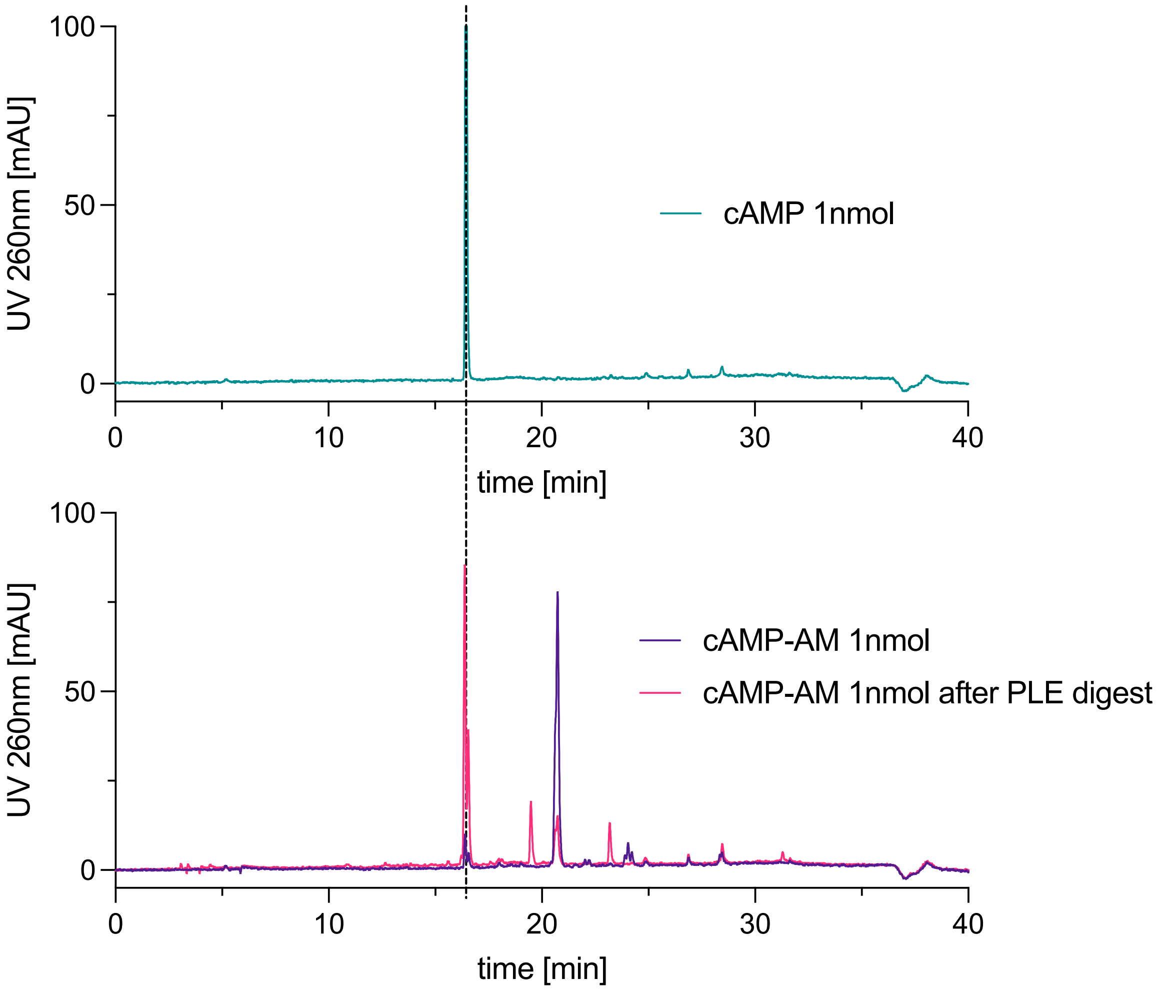

HPLC analysis of cAMP standard (1 nmol; turquoise, top panel), as well as commercially available cAMP-AM (1nmol; violet, bottom panel) before and after digestion with porcine liver esterase (PLE; pink). The retention time of standard cAMP is additionally indicated by a dashed line. Compounds were separated by reversed-phase-HPLC as described in Materials and Methods. A representative experiment out of 3 is shown. Source data are provided as a Source Data file.

Supplementary Fig. 12: Lack of expression of HN1L/JPT2 in Neuro2A *Hn1l/Jpt2*$^{-/-}$ cells

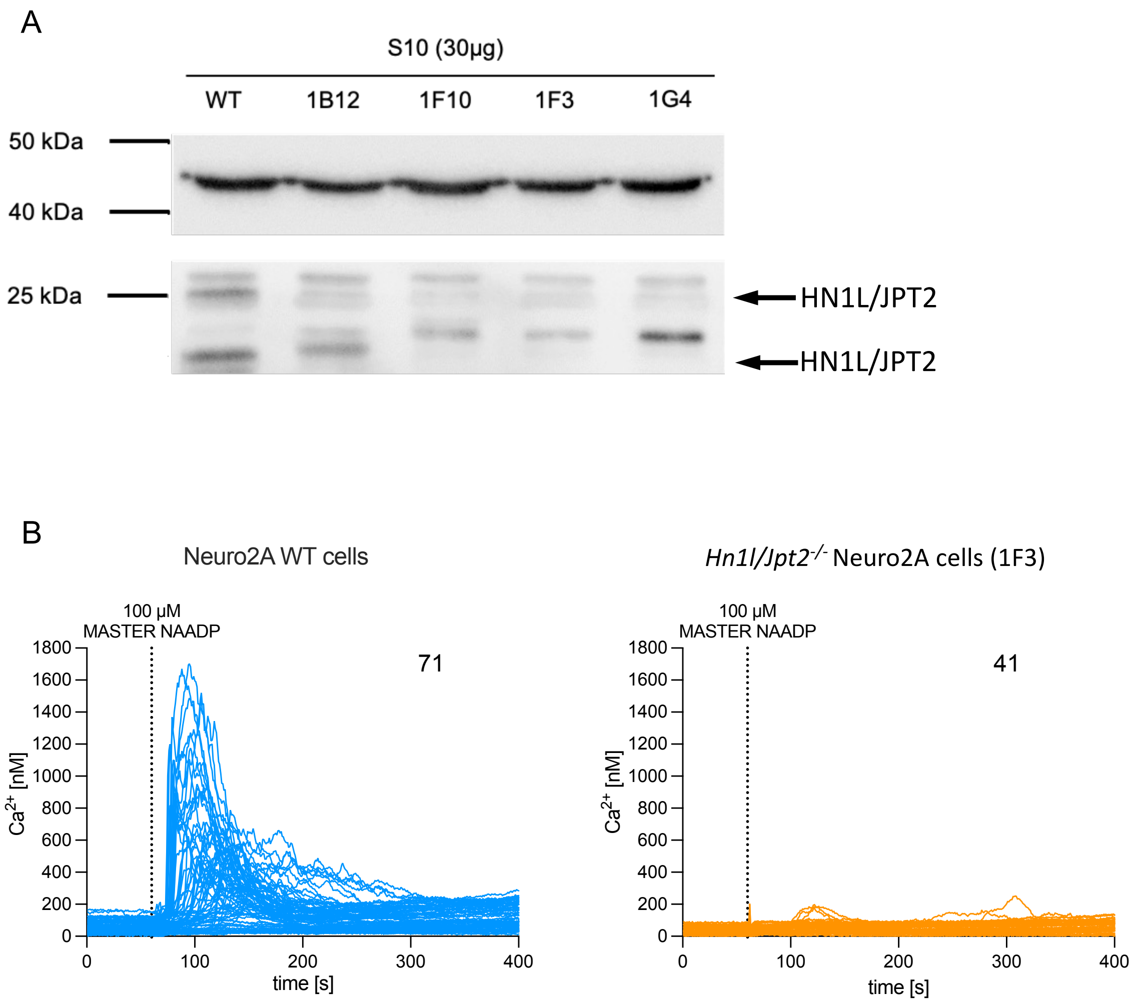

(**A**) Western blot analysis of HN1L/JPT2 in WT compared to four different Neuro2a *Hn1l*$^{-/-}$ clones was carried out using anti-HN1L/JPT2 antibody orb1412 (Biorbyt). Protein amount was 30 µg of S10 protein per lane. ***β***-Actin was used as a loading control (upper part of gel). Lack of HN1L/JPT2 expression was detected for *Hn1l*$^{-/-}$ clones 1F10, 1F3 and 1G4; for 1B12 the situation is unclear and thus, 1G12 cells were not further used (lower part of gel, n = 3 independent experiments). For further analysis the clones 1G4 and 1F3 were used. (**B**) Global Ca^{2+} tracings for Neuro2A WT (same data set as in figure 6F) compared to *Hn1l*$^{-/-}$ clone 1F3. The data is displayed as mean ± SEM; Neuro2A WT MASTER-NAADP, n = 71 cells; Neuro2A *Hnl1*$^{-/-}$ clone 1F3 MASTER-NAADP, n = 41 cells. Source data are provided as a Source Data file.

Supplementary Fig. 13: Comparison of HN1L/JTP2 protein levels in different cell types

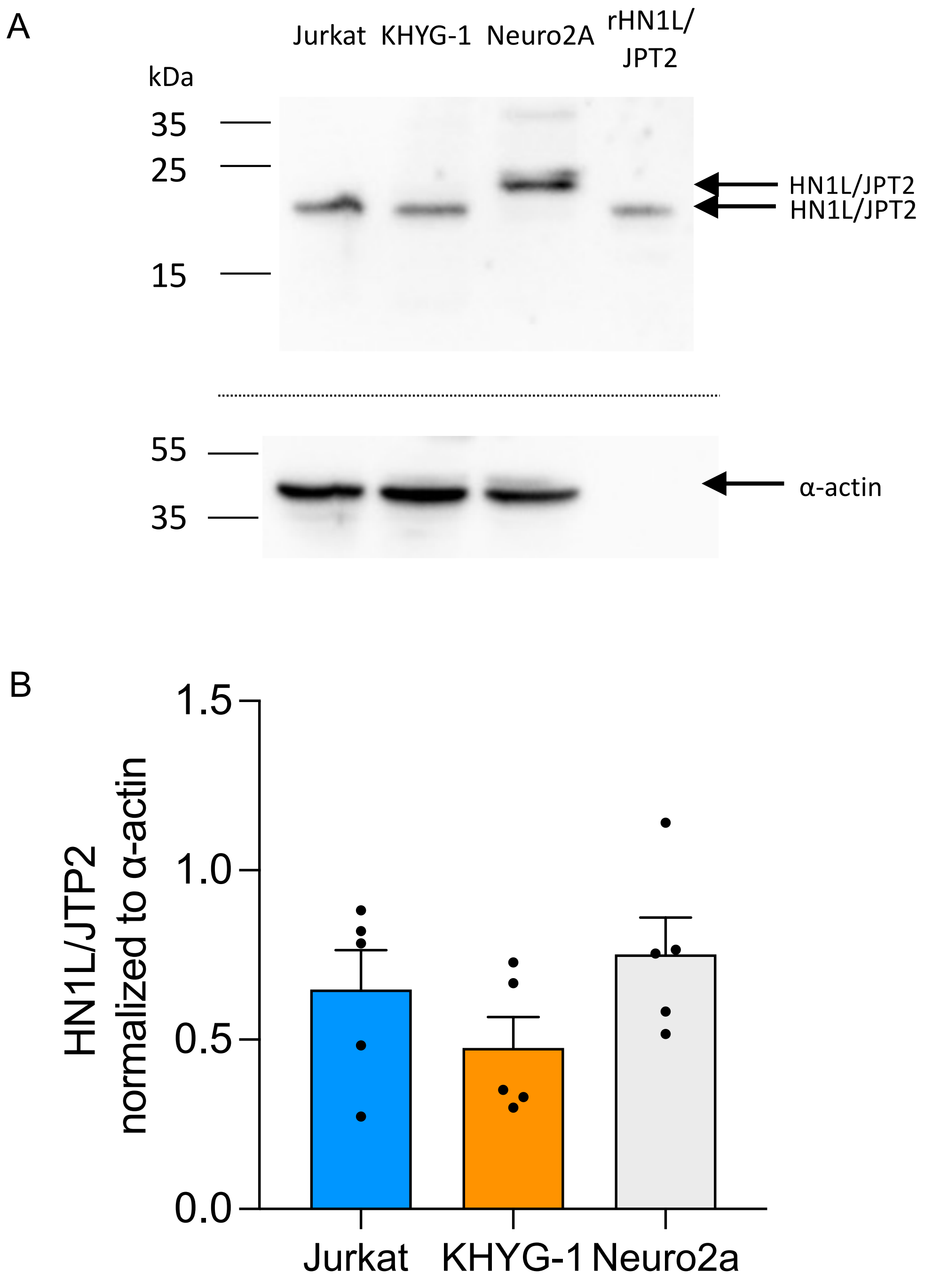

(**A**) Western blot analysis of HN1L/JPT2 in WT Jurkat, KHYG-1 and Neuro2a cells was performed using anti-HN1L/JPT2 antibody HPA041888 (Atlas Antibodies). Protein amount was 100 µg of S10 protein per lane. α-Actin was used as a loading control (lower part of gel). 8 ng of recombinant HN1L/JPT2 was used as positive control. Shown is a representative western blot from 5 independent experiments. (**B**) Analysis of HN1L/JPT2 expression normalized to α-actin. The data are displayed as mean ± SEM (n=5). Source data and exact *p* values are provided as a Source Data file.

Supplementary Methods

Compound synthesis and characterization

1,2-*O*-*Iso*propylidene-D-xylofuranose (2). To a 0 °C cooled solution of 20 g (0.13 mol, 1.0 eq.) D-xylose **1** in 600 mL dry acetone 2.0 g (13 mmol, 0.10 equiv.) copper sulfate was added and 20 mL (37 g, 0.38 mol, 2.8 eq.) conc. sulfuric acid was added dropwise. The reaction mixture was stirred for 3 h at room temperature. After the suspension was filtered, the residue was washed with acetone, and the filtrate neutralized with 25 % ammonia solution, the solvent was removed under reduced pressure. The residue was dissolved in 250 mL of 0.2 % HCl solution and the reaction solution was stirred at room temperature for 6 h. The reaction mixture was neutralized with saturated sodium bicarbonate solution, the aqueous layer was extracted twice with CH_2Cl_2, and the H_2O was removed under reduced pressure. The crude product was purified several times by column chromatography on silica gel (CH_2Cl_2/CH_3OH 50:1 v/v → CH_2Cl_2/CH_3OH 10:1 v/v), (CH_2Cl_2/CH_3OH 100:1 v/v → CH_2Cl_2/CH_3OH 10:1 v/v). Yield: 25 g (0.13 mol, 99 %) as a colorless oil. ^{1}H-NMR: (600 MHz, DMSO-d_6): δ 5.80 (d, J = 3.7 Hz, 1H), 5.14 (d, J = 4.8 Hz, 1H), 4.61 (t, J = 5.7 Hz, 1H), 4.37 (d, J = 3.7 Hz, 1H), 4.01-3.92 (m, 2H), 3.60 (ddd, J = 11.3 Hz, J = 5.6 Hz, J = 5.6 Hz, 1H), 3.55-3.44 (m, 1H), 1.37 (s, 3H), 1.22 (s, 3H); ^{13}C-NMR: (151 MHz, $CDCl_3$): δ 110.6, 104.7, 85.5, 81.8, 73.9, 59.3, 27.2, 26.6; IR: (ATR) $\tilde{\nu}$ [cm^{-1}]: 3395, 2986, 2937, 1455, 1375, 1294, 1253, 1213, 1163, 1068, 1004, 903, 885, 857, 825, 787, 738, 633, 565, 509, 455, 425; HRMS (ESI, m/z): [M+Na]$^+$ calcd. for $C_8H_{14}O_5$, 213.0733; found, 213.0730.

1,2-*O*-*Iso*propylidene-5-*O*-benzoyl-D-xylofuranose (3). In a nitrogen atmosphere, 1.1 g (6.0 mmol, 1.0 eq.) 1,2-*O*-isopropylidene-D-xylofuranose **2** was dissolved in 20 mL dry CH_2Cl_2. Under ice cooling, 1.3 mL (0.91 g, 9.0 mmol, 1.5 eq.) dry Et_3N was added and 0.76 mL (0.93 g, 6.6 mmol, 1.1 eq.) benzoyl chloride was added slowly. The reaction was stirred at 0 °C for 1 h, followed by addition of saturated sodium bicarbonate solution. The organic layer was washed twice with saturated sodium bicarbonate solution and dried with Na_2SO_4. The solvent was removed under reduced pressure. The crude product was purified by column chromatography on silica gel (PE/EtOAc 4:1 v/v → PE/EA 3:1 v/v). Yield: 1.6 g (5.5 mmol, 92 %) as a colorless oil. ^{1}H-NMR: (500 MHz, DMSO-d_6): δ 8.01-7.95 (m, 2H), 7.71-7.64 (m, 1H), 7.58-7.50 (m, 2H), 5.89 (d, J = 3.7 Hz, 1H), 5.49 (d, J = 5.0 Hz, 1H), 4.49-4.43 (m, 2H), 4.39-4.32 (m, 2H), 4.13 (dd, J = 5.0 Hz, J = 2.4 Hz, 1H), 1.40 (s, 3H), 1.25 (s, 3H); ^{13}C-NMR: (125 MHz, DMSO-d_6): δ 165.6, 133.4, 129.2, 128.8, 110.7, 104.6, 85.0, 78.2, 73.7, 63.1, 26.7, 26.0; IR: (ATR) $\tilde{\nu}$ [cm^{-1}]: 3421, 2991, 2947, 1714, 1600, 1585, 1449, 1378, 1355, 1345, 1318, 1272, 1257, 1213, 1178, 1162, 1122, 1108, 1091, 1070, 1059, 1014, 996, 977, 942, 885, 854, 837, 813, 748, 707, 686, 659, 638, 613, 578, 548, 511, 447, 422; HRMS (ESI, m/z): [M+Na]$^+$ calcd. for $C_{16}H_{18}O_6$, 317.0996; found, 317.0998.

1-*O*-Methyl-5-*O*-benzoyl-D-xylofuranose (4). In a nitrogen atmosphere, 2.8 g (9.7 mmol, 1.0 eq.) compound **3** was dissolved in 60 mL dry CH_3OH and 0.60 g (2.4 mmol, 0.2 eq.) iodine was added. The reaction was heated to reflux for 4 h and then saturated sodium thiosulfate solution was added. The aqueous layer was extracted five times with CH_2Cl_2 and the combined organic layers were washed once with saturated sodium chloride solution. The organic layer was dried with Na_2SO_4 and the solvent was removed under reduced pressure. The crude product was purified by column chromatography on silica

gel (PE/EtOAc 1:1 v/v). Yield: 2.2 g (8.3 mmol, 86 %) as a colorless oil. ^{1}H-NMR: (500 MHz, DMSO-*d*$_6$): *δ* 8.01-7.94 (m, 4H), 7.70-7.63 (m, 2H), 7.57-7.51 (m, 4H), 5.40 (d, *J* = 4.4 Hz, 1H), 5.37 (d, *J* = 5.0 Hz, 1H), 5.15 (d, *J* = 5.1 Hz, 1H), 4.91 (d, *J* = 6.5 Hz, 1H), 4.80 (d, *J* = 4.2 Hz, 1H), 4.68 (d, *J* = 1.4 Hz, 1H), 4.49-4.38 (m, 3H), 4.36-4.25 (m, 3H), 4.13 (q, *J* = 5.7 Hz, 1H), 4.06 (td, *J* = 5.3 Hz, *J* = 2.7 Hz, 1H), 3.93-3.86 (m, 2H), 3.32 (s, 3H), 3.24 (s, 3H); ^{13}C-NMR: (125 MHz, DMSO-*d*$_6$): *δ* 165.7, 165.7, 133.3, 133.3, 129.8, 129.7, 129.1, 129.2, 128.7, 128.7, 109.6, 102.3, 80.8, 79.1, 77.1, 75.8, 75.6, 74.6, 65.0, 64.3, 56.0, 53.6; IR: (ATR) $\tilde{\nu}$ [cm^{-1}]: 3436, 3064, 2936, 2836, 1715, 1602, 1584, 1451, 1400, 1316, 1270, 1191, 1178, 1096, 1071, 1038, 1025, 976, 883, 807, 773, 736, 708, 687, 607, 533, 432; HRMS (ESI, m/z): [M+Na]$^{+}$ calcd. for $C_{13}H_{16}O_6$, 291.0839; found, 291.0840.

1-*O*-Methyl-3-deoxy-3-fluoro-5-*O*-benzoyl-D-xylofuranose (5). In a nitrogen atmosphere, 0.24 g (0.90 mmol, 1.0 eq.) 1.2-*O*-methyl-5-*O*-benzoyl-D-xylofuranose **4** was dissolved in 6.0 mL dry CH_2Cl_2. At -78 °C, 0.18 mL (0.22 g, 1.4 mmol, 1.5 eq.) diethylaminosulfur trifluoride (DAST) was added dropwise to the solution. The reaction was stirred for 20 h, slowly warming to room temperature. After addition of saturated sodium bicarbonate solution, the aqueous layer was extracted four times with CH_2Cl_2. The combined organic layers were washed once with water and dried with Na_2SO_4. Under reduced pressure the solvent was removed. The crude product was purified by column chromatography on silica gel (PE/EtOAc 4:1 v/v → PE/EtOAc 3:1 v/v → EtOAc). Yield: 0.11 g (0.39 mmol, 43 %) as colorless oil. ^{1}H-NMR: (600 MHz, DMSO-*d*$_6$): *δ* 8.01-7.98 (m, 2H), 7.96-7.94 (m, 2H), 7.70-7.66 (m, 2H), 7.57-7.53 (m, 4H), 5.70 (d, *J* = 5.5 Hz, 1H), 5.15-5.03 (m, 2H), 4.98-4.87 (m, 2H), 4.78-4.76 (m, 1H), 4.49-4.30 (m, 6H), 4.10-4.02 (m, 2H), 3.32 (s, 3H), 3.23 (s, 3H); ^{13}C-NMR: (151 MHz, DMSO-*d*$_6$): *δ* 165.5, 165.4, 133.6, 133.5, 129.5, 129.2, 129.2, 128.8, 128.8, 108.2, 102.5, 91.8 (d, *J* = 187.8 Hz), 90.6 (d, *J* = 185.1 Hz), 79.9 (d, *J* = 25.9 Hz), 78.2 (d, *J* = 25.9 Hz), 73.2 (d, *J* = 16.2 Hz), 71.6 (d, *J* = 11.3 Hz), 64.1 (d, *J* = 10.6 Hz), 64.0 (d, *J* = 6.2 Hz), 55.0, 54.9; ^{19}F-NMR: (565 MHz, DMSO-*d*$_6$): *δ* -192.73 (m), -209.03 (m); IR: (ATR) $\tilde{\nu}$ [cm^{-1}]: 3468, 2939, 2838, 1718, 1602, 1584, 1492, 1451, 1413, 1382, 1315, 1269, 1178, 1100, 1068, 1038, 1024, 976, 950, 868, 806, 779, 755, 709, 687, 675, 602, 559, 535, 476; HRMS (ESI, m/z): calcd. for [M+Na]$^{+}$ $C_{13}H_{15}FO_5$, 293.0796; found, 293.0794.

1,2-Di-*O*-acetyl-3-deoxy-3-fluoro-5-*O*-benzoyl-D-xylofuranose (6). To a solution of 0.37 g (1.4 mmol, 1.0 eq.) 1-*O*-methyl-3-deoxy-3-fluoro-5-*O*-benzoyl-D-xylo-furanose **5** in 7.0 mL HOAc, 0.61 mL (0.66 g, 6.5 mmol, 4.7 eq.) acetic acid anhydride and 0.35 mL (0.63 g, 6.5 mmol, 4.7 eq.) conc. sulfuric acid were added. The reaction solution was stirred for 19 h at room temperature. The reaction solution was cooled to 0 °C and saturated sodium hydrogen carbonate solution was added. The aqueous layer was extracted four times with CH_2Cl_2. The combined organic layers were dried with Na_2SO_4 and the solvent was removed under reduced pressure. The crude product was purified by column chromatography on silica gel (PE/EtOAc 3:1 v/v). Yield: 0.43 g (1.3 mmol, 93 %) as colorless oil. ^{1}H-NMR: (600 MHz, DMSO-*d*$_6$): *δ* 8.06-8.03 (m, 2H), 8.00-7.97 (m, 2H), 7.72-7.67 (m, 2H), 7.59-7.53 (m, 4H), 6.41 (d, *J* = 4.8 Hz, 1H), 6.10 (s, 1H), 5.55 (dt, *J* = 52.0 Hz, *J* = 4.2 Hz, 1H), 5.45-5.34 (m, 2H), 5.22 (dt, *J* = 25.1 Hz, *J* = 5.1 Hz, 1H), 4.77 (dtd, *J* = 26.8 Hz, *J* = 3.9 Hz, *J* = 1.3 Hz, 1H), 4.67 (dq, *J* = 20.8 Hz, *J* = 3.9 Hz, 1H), 4.60 (dd, *J* = 12.3 Hz, *J* = 3.7 Hz, 1H), 4.47-4.45 (m, 2H), 4.42 (dd, *J* = 12.3 Hz, *J* = 4.2 Hz, 1H), 2.15-2.11 (m, 6H), 2.08 (s, 3H), 1.87 (s, 3H); ^{13}C-NMR: (151 MHz, DMSO-*d*$_6$): *δ* 169.6,

169.5, 169.3, 169.0, 165.3, 165.2, 133.6, 129.3, 129.3, 129.2, 128.8, 128.8, 98.0, 93.1, 89.4 (d, *J* = 189.8 Hz), 88.6 (d, *J* = 186.5 Hz), 81.7 (d, *J* = 27.1 Hz), 80.8 (d, *J* = 24.8 Hz), 74.5 (d, *J* = 13.5 Hz), 70.9 (d, *J* = 15.8 Hz), 63.6 (d, *J* = 11.8 Hz), 63.1 (d, *J* = 7.2 Hz), 20.9, 20.5, 20.3, 20.2; ^{19}F-NMR: (565 MHz, DMSO-d_6): *δ* -194.32 (m), -206.90 (m); IR: (ATR) $\tilde{\nu}$ [cm^{-1}]: 2955, 1746, 1721, 1602, 1584, 1452, 1372, 1315, 1270, 1211, 1178, 1108, 1070, 1045, 1024, 1009, 967, 935, 897, 864, 805, 782, 736, 709, 688, 628, 601, 544, 514, 490, 446; HRMS (ESI, m/z): [M+Na]$^+$ calcd. for $C_{16}H_{17}FO_7$, 363.0851; found, 363.0850.

9-(2’-*O*-Acetyl-3’-deoxy-3’-fluoro-5’-*O*-benzoyl-*β*-D-xylofuranosyl)-N6-benzoyladenine (7). In a nitrogen atmosphere, 0.43 g (1.8 mmol, 1.5 eq.) *N*-benzoyladenine was suspended in 3 mL CH_2Cl_2 and 1.2 mL (0.97 g, 4.8 mmol, 4.0 eq.) bis(trimethylsilyl)acetamide (BSA) was added slowly. The reaction mixture was heated at reflux for 60 minutes, forming a solution. After cooling the mixture, a solution of 0.41 g (1.2 mmol, 1.0 eq.) 1,2-di-*O*-acetyl-3-deoxy-3-fluoro-5-*O*-benzoyl-D-xylo-furanose **6** in 2 mL CH_2Cl_2 was added. At 0 °C 0.86 mL (1.1 g, 4.8 mmol, 4.0 equiv.) TMSOTf was added. The reaction solution was heated for 5.5 h to reflux. After adding saturated sodium bicarbonate solution to the reaction solution at 0 °C, the aqueous layer was extracted three times with CH_2Cl_2. The combined organic layers were washed twice with saturated sodium hydrogen carbonate solution and dried with Na_2SO_4. The solvent was removed under reduced pressure. The crude product was purified by column chromatography on silica gel (PE/EtOAc 1:5 v/v). Yield: 0.38 g (0.73 mmol, 61 %) of a yellowish solid. ^{1}H-NMR: (600 MHz, DMSO-d_6): *δ* 8.67 (s, 1H), 8.46 (s, 1H), 8.05-8.01 (m, 4H), 7.70-7.62 (m, 2H), 7.57-7.53 (m, 4H), 6.44 (d, *J* = 7.0 Hz, 1H), 6.31 (ddd, *J* = 18.2 Hz, *J* = 7.1 Hz, *J* = 4.8 Hz, 1H), 5.77 (ddd, *J* = 53.1 Hz, *J* = 4.9 Hz, *J* = 2.3 Hz, 1H), 4.80-4.59 (m, 3H), 2.10 (s, 3H); ^{13}C-NMR: (151 MHz, DMSO-d_6): *δ* 169.9, 165.9, 152.4, 152.1, 151.2, 144.3, 134.1, 133.0, 129.8, 129.7, 129.2, 129.0, 128.9, 126.4, 89.7 (d, *J* = 187.0 Hz), 85.3, 80.7 (d, *J* = 24.6 Hz), 27.2 (d, *J* = 15.4 Hz), 63.6 (d, *J* = 8.9 Hz), 20.8; ^{19}F-NMR: (565 MHz, DMSO-d_6): *δ* -198.93 (ddd, *J* = 53.2 Hz, *J* = 24.8 Hz, *J* = 18.2 Hz); IR: (ATR) $\tilde{\nu}$ [cm^{-1}]: 3064, 2943, 1747, 1718, 1639, 1602, 1582, 1509, 1485, 1451, 1371, 1314, 1267, 1217, 1177, 1092, 1068, 1025, 917, 891, 797, 708, 643, 612, 564; HRMS (ESI, m/z): [M+H]$^+$ calcd. for $C_{26}H_{22}FN_5O_6$, 520.1627; found, 520.1625.

3’-Deoxy-3’-fluoro-adenosine (8). A suspension of 0.99 g (1.9 mmol, 1.0 eq.) 9-(2'-*O*-acetyl-3'-deoxy-3'-fluoro-5'-*O*-benzoyl-*β*-D-xylofuranosyl)-*N*6-benzoyladenine **7** in 22 mL 7 N ammonia in CH_3OH was stirred in a sealed tube at 70 °C for 28 h. The solvent was removed under reduced pressure and the crude product was purified by column chromatography on silica gel (EtOAc/CH_3OH 20:1 v/v→ EtOAc/CH_3OH 15:1 v/v). Yield: 0.43 g (1.6 mmol, 84 %) of a colorless crystalline solid. ^{1}H-NMR: (600 MHz, CH_3OD): *δ* 8.28 (s, 1H), 8.18 (s, 1H), 6.00 (d, *J* = 7.9 Hz, 1H), 5.13 (dd, *J* = 54.5 Hz, *J* = 4.3 Hz, 1H), 4.98 (ddd, *J* = 25.2 Hz, *J* = 8.0 Hz, *J* = 4.2 Hz, 1H), 4.44 (dt, *J* = 27.7 Hz, *J* = 2.6 Hz, 1H), 3.86 (ddd, *J* = 12.6 Hz, *J* = 2.5 Hz, *J* = 2.5 Hz 1H), 3.80 (ddd, *J* = 12.7 Hz, *J* = 2.3 Hz, *J* = 2.3 Hz, 1H); ^{13}C-NMR: (151 MHz, CH_3OD): *δ* 157.7, 153.5, 150.1, 142.1, 121.2, 94.5 (d, *J* = 187.1 Hz), 90.4, 86.4 (d, *J* = 31.4 Hz), 74.5 (d, *J* = 22.1 Hz), 63.1 (d, *J* = 11.6 Hz); ^{19}F-NMR: (565 MHz, CH_3OD): *δ* -199.98 (ddd, *J* = 54.3 Hz, *J* = 26.8 Hz, *J* = 26.6 Hz); IR: (ATR) $\tilde{\nu}$ [cm^{-1}]: 3301, 3162, 3062, 2920, 2363, 2218, 2194, 2181, 2160, 2030, 2022, 2005, 1978, 1968, 1941, 1894, 1844, 1830, 1734, 1717, 1684,

1653, 1637, 1604, 1579, 1558, 1541, 1521, 1507, 1489, 1473, 1457, 1447, 1437, 1425, 1374, 1335, 1293, 1227, 1124, 1078, 1030, 982, 897, 873, 795, 743, 731, 698, 668, 646, 632, 573, 564, 532, 485, 457, 438, 420, 408, 396, 389; HRMS (ESI, m/z): $[M+H]^+$ calcd. for $C_{10}H_{12}FN_5O_3$, 270.0997; found, 270.1000.

3'-Deoxy-3'-fluoro-5'-*O*-tertbutyldimethylsilyl-adenosine (9). In a nitrogen atmosphere, 0.19 g (0.69 mmol, 1.0 eq.) 3'-deoxy-3' fluoro-adenosine **8** was dissolved in 11 mL dry pyridine and 0.16 g (1.0 mmol, 1.5 eq.) of *tert*-butyl-dimethylsilylchloride was added. The reaction mixture was stirred for 24 h at room temperature. The solvent was removed under reduced pressure, the residue was solved in CH_2Cl_2 and washed once with saturated sodium bicarbonate solution and once with sodium chloride solution. The combined organic layers were dried with Na_2SO_4. The solvent was removed under reduced pressure and the crude product was purified by column chromatography on silica gel (CH_2Cl_2/CH_3OH 50:1 v/v→ CH_2Cl_2/CH_3OH 30:1 v/v). Yield: 0.19 g (0.49 mmol, 71 %) of a colorless solid. ^{1}H-NMR: (600 MHz, DMSO-d_6): δ 8.30 (s, 1H), 8.15 (s, 1H), 7.34 (s, 2H), 5.98 (d, J = 6.3 Hz, 1H), 5.95 (d, J = 7.6 Hz, 1H), 5.02 (ddd, J = 54.5 Hz, J = 4.2 Hz, J = 1.2 Hz, 1H), 5.01-4.92 (m, 1H), 4.94 (dddd, J = 26.2 Hz, J = 4.7 Hz, J = 4.7 Hz, J = 1.2 Hz, 1H), 3.87-3.80 (m, 2H), 0.88 (s, 9H), 0.07 (s, 3H), 0.06 (s, 3H); ^{13}C-NMR: (151 MHz, DMSO-d_6): δ 156.6, 153.3, 150.1, 139.7, 119.5, 92.8 (d, J = 183.3 Hz), 86.4, 83.3 (d, J = 26.8 Hz), 72.5 (d, J = 16.2 Hz), 62.9 (d, J = 11.2 Hz), 26.3, 18.5, -5.5; ^{19}F-NMR: (565 MHz, DMSO-d_6): δ -198.60 (ddd, J = 53.5 Hz, J = 28.7 Hz, J = 25.9 Hz); IR: (ATR) $\tilde{\nu}$ [cm^{-1}]: 3339, 3215, 3150, 2950, 2928, 2882, 2855, 1635, 1600, 1577, 1508, 1485, 1417, 1387, 1361, 1333, 1323, 1303, 1241, 1207, 1178, 1127, 1105, 1092, 1036, 995, 966, 897, 875, 829, 796, 785, 709, 648, 658, 631, 621, 574, 559, 545, 527, 486, 439, 399; HRMS (ESI, m/z): $[M+H]^+$ calcd. for $C_{16}H_{26}FN_5O_3Si$, 384.1862; found, 384.1860.

4-(Hydroxymethyl)phenylheptanoate (11). General procedure A with 3.2 g (26 mmol, 1.2 eq.) 4-hydroxybenzyl alcohol **10**, 4.4 mL (3.2 g, 26 mmol, 1.2 eq.) Et_3N dissolved in 40 mL dry THF at 0 °C. A solution of 4.9 g (22 mmol, 1.0 eq.) heptanoyl chloride in 10 mL dry THF was added. The reaction was stirred for 2 h at 0 °C. The crude product was purified by column chromatography on silica gel (PE/EtOAc 4:1 v/v → PE/EtOAc 4:2 v/v). Yield: 4.45 g (18.9 mmol, 87 %) as a colorless oil. ^{1}H-NMR:(500 MHz, DMSO-d_6): δ 7.36-7.31 (m, 2H), 7.06-7.02 (m, 2H), 5.20 (t, J = 5.7 Hz, 1H), 4.48 (d, J = 5.7 Hz, 2H), 2.96 (t, J = 7.4 Hz, 2H), 1.62 (quint, J = 7.4 Hz, 2H), 1.40-1.22 (m, 6H), 0.91-0.84 (m, 3H); ^{13}C-NMR: (126 MHz, DMSO-d_6): δ 172.3, 149.5, 140.4, 127.8, 121.7, 62.7, 33.8, 31.2, 28.4, 22.3, 24.7, 14.3; IR: (ATR) $\tilde{\nu}$ [cm^{-1}]: 3392, 2955, 2929, 2859,1754, 1606, 1507, 1458, 1417, 1365, 1195, 1163, 1101, 1042, 1014, 941, 916, 847, 811, 760, 728, 559, 503, 439, 398; HRMS (ESI, m/z): $[M+Na]^+$ calcd. for $C_{14}H_{20}O_3$, 259.1298; found, 259.1270.

***N,N*-Di*iso*propylamino-bis(4-heptanoyloxybenzyl)phosphoramidite (12).** General procedure B with 0.40 g (2.0 mmol, 1.0 eq.) dichloro-*N,N*-di*iso*propylphosphoramidite dissolved in 5 mL THF, 1.0 g (4.4 mmol, 2.2 eq.) 4-(hydroxymethyl)phenylheptanoate **11** and 0.64 mL (0.47 g, 4.6 mmol, 2.3 eq.) Et_3N in 13 mL THF. The crude product was purified by column chromatography on silica gel (PE/EtOAc 5:1). Yield: 1.1 g (1.8 mmol, 92 %) as a colorless oil. ^{1}H-NMR:(500 MHz, $CDCl_3$): δ 7.36-7.33 (m, 4H),

7.05-7.01 (m, 4H), 4.74 (dd, 2H, *J* = 12.6 Hz, *J* = 8.1 Hz), 4.67 (dd, 2H, *J* = 12.6 Hz, *J* = 8.6 Hz), 3.73-3.64 (m, 2H), 2.54 (t, *J* = 7.5 Hz, 4H), 1.80-1.69 (m, 4H), 1.46-1.38 (m, 4H), 1.35-1.30 (m, 8H), 1.20 (d, *J* = 6.8 Hz, 12H), 0.93-0.89 (m, 6H); ^{13}C-NMR: (126 MHz, $CDCl_3$): δ 172.8, 150.3, 137.4, 128.4, 121.8, 65.3 (d, *J* = 17.5 Hz), 43.5 (d, *J* = 10.8 Hz), 34.8, 31.9, 31.9, 29.2, 25.3, 25.0 (d, *J* = 7.2 Hz), 22.9, 14.4; IR: (ATR) $\tilde{\nu}$ [cm^{-1}]: 2961, 2929, 2860, 1758, 1608, 1507, 1459, 1417, 1396, 1364, 1296, 1196, 1163, 1138, 1102, 1052, 1026, 1005, 972, 942, 915, 851, 802, 752, 640, 521, 504, 470, 437, 417, 407, 398.

2'-(Bis-*O*-(4-heptanoyloxybenzyl))-(5'-*O*-*tert*-butyldimethylsilyl-3'-deoxy-3'-fluoro-adenosine)-2'-phosphate (13). Under nitrogen atmosphere, 0.35 g (0.90 mmol, 1.0 eq.) 3'-deoxy-3'-fluoro-5'-*O*-*tert*-butyl-dimethylsilyladenosine **9** was suspended in 17 mL dry CH_2Cl_2. After 0.70 g (1.2 mmol, 1.3 eq.) *N,N*-di*iso*propylamino-bis-(4-heptanoyloxy-benzyl)phosphoramidite **12** and 0.21 g (1.1 mmol, 1.2 eq.) pyridinium trifluoroacetate were added the reaction mixture was stirred at room temperature for 50 minutes. After 0.27 mL (1.3 mmol, 1.5 eq.) of *tert*-butylhydroxyperoxide (5.0 M in decane) were added under ice cooling the reaction mixture was stirred for 1 h at room temperature. Under reduced pressure, the solvent was removed and the residue was purified several times by column chromatography on silica gel (CH_2Cl_2/CH_3OH 50:1 v/v). Yield: 0.71 g (0.78 mmol, 87 %) of a colorless solid. ^{1}H-NMR: (600 MHz, DMSO-*d*$_6$): δ 8.31 (s, 1H), 8.14 (s, 1H), 7.43 (s, 2H), 7.30-7.26 (m, 2H), 7.15-7.06 (m, 2H), 7.10-7.06 (m, 2H), 7.05-7.01 (m, 2H), 6.25 (d, *J* = 7.3 Hz, 1H), 5.79-5.70 (m, 1H) 5.30 (ddd, *J* = 53.4 Hz, *J* = 4.3 Hz, *J* = 1.4 Hz, 1H), 4.97 (dd, *J* = 11.9 Hz, *J* = 8.0 Hz, 1H), 4.86 (dd, *J* = 11.9 Hz, *J* = 8.4 Hz, 1H), 4.78 (dd, *J* = 12.3 Hz, *J* = 7.9 Hz, 2H), 4.42 (dddd, *J* = 25.8 Hz, *J* = 4.2 Hz, *J* = 4.2 Hz, *J* = 1.4 Hz, 1H), 3.88 (m, 2H), 2.57 (t, *J* = 7.4 Hz, 4H), 1.67-1.59 (m, 4H), 1.39-1.33 (m, 4H), 1.32-1.26 (m, 8H), 0.91-0.86 (m, 6H), 0.85 (s, 9H), 0.05 (s, 3H), 0.04 (s, 3H); ^{13}C-NMR: (151 MHz, DMSO-*d*$_6$): δ 171.8, 171.7, 156.2, 153.0, 150.5, 149.5, 139.0, 132.9 (d, *J* = 7.1 Hz), 132.7 (d, *J* = 7.1 Hz), 129.1, 129.0, 121.9, 121.8, 119.0, 90.4 (d, *J* = 187.8 Hz), 84.4, 82.9 (d, *J* = 22.2 Hz), 75.6 (dd, *J* = 15.2 Hz, *J* = 4.9 Hz), 68.6, (d, *J* = 5.5 Hz), 68.4 (d, *J* = 4.9 Hz), 62.0 (d, *J* = 11.2 Hz), 33.4, 30.9, 28.1, 25.7, 24.3, 22.0, 17.9, 13.9, -5.6; ^{19}F-NMR: (565 MHz, DMSO-*d*$_6$): δ -198.51 (ddd, *J* = 53.4 Hz, *J* = 25.5 Hz, *J* = 20.5 Hz); ^{31}P-NMR: (243 MHz, DMSO-*d*$_6$): δ -2.08; IR: (ATR) $\tilde{\nu}$ [cm^{-1}]: 3326, 3176, 2954, 2929, 2857, 1756, 1644, 1597, 1579, 1509, 1470, 1421, 1365, 1330, 1255, 1199, 1167, 1136, 1104, 1025, 1002, 920, 832, 798, 778, 714, 685, 649, 567, 500, 400; HRMS (ESI, m/z): $[M+H]^+$ calcd. for $C_{44}H_{63}FN_5O_{10}PSi$, 900.4139; found, 900.4135.

2'-(Bis-*O*-(4-heptanoyloxybenzyl))-(3'-deoxy-3'-fluoro-adenosine)-2'-phosphate (14). In a nitrogen atmosphere, 0.74 g (0.82 mmol, 1.0 eq.) compound **13** was dissolved in 10 mL dry CH_2Cl_2, followed by the slow addition of 0.82 mL (0.81 g, 5.0 mmol, 5.2 eq.) triethylamine-trihydrofluoride. After the reaction mixture was stirred for 19 h at room temperature, silica gel was added to the reaction and the solvent was removed under reduced pressure. The residue was purified by column chromatography on silica gel (CH_2Cl_2/CH_3OH 30:1 v/v → CH_2Cl_2/CH_3OH 20:1 v/v). Yield: 0.52 g (0.67 mmol, 81 %) of a colorless solid. ^{1}H-NMR: (600 MHz, DMSO-*d*$_6$): δ 8.41 (s, 1H), 8.15 (s, 1H), 7.50 (s, 2H), 7.29-7.25 (m, 2H), 7.15-7.11 (m, 2H), 7.10-7.06 (m, 2H), 7.06-7.02 (m, 2H), 6.27 (d, *J* = 7.7 Hz, 1H), 5.89 (dd, *J* = 7.3 Hz, *J* = 4.6 Hz), 5.71 (ddd, *J* = 22.3 Hz, *J* = 8.0 Hz, *J* = 4.0 Hz 1H) 5.35 (ddd, *J* = 53.8 Hz, *J* = 4.7 Hz, 1H),

4.96 (dd, *J* = 11.9 Hz, *J* = 8.0 Hz, 1H), 4.85 (dd, *J* = 11.8 Hz, *J* = 8.3 Hz, 1H), 4.78-4.71 (m, 2H), 4.50-4.41 (m, 1H), 3.74-3.64 (m, 2H), 2.57 (dt, *J* = 7.5 Hz, *J* = 1.8 Hz, 4H), 1.63 (p, *J* = 7.5 Hz, 4H), 1.40-1.19 (m, 12H), 0.92-0.84 (m, 6H); ^{13}C-NMR: (151 MHz, DMSO-*d*$_6$): *δ* 171.8, 171.7, 156.4, 152.7, 150.5, 149.0, 140.1, 132.9 (d, *J* = 8.0 Hz), 132.7 (d, *J* = 8.0 Hz), 129.2, 129.0, 121.9, 121.9, 119.5, 91.5 (d, *J* = 186.2 Hz), 85.2, 84.2 (d, *J* = 20.2 Hz), 75.5 (dd, *J* = 15.7 Hz, *J* = 5.4 Hz), 68.6, (d, *J* = 5.3 Hz), 68.4 (d, *J* = 5.5 Hz), 62.7 (d, *J* = 10.9 Hz), 33.4, 30.9, 28.0, 24.2, 22.0, 14.0; ^{19}F-NMR: (565 MHz, DMSO-*d*$_6$): *δ* -197.10 (ddd, *J* = 53.2 Hz, *J* = 29.7 Hz, *J* = 23.0 Hz); ^{31}P-NMR: (243 MHz, DMSO-*d*$_6$): *δ* -2.11; IR: (ATR) $\tilde{\nu}$ [cm^{-1}]: 3328, 3173, 2929, 2858, 1754, 1644, 1598, 1509, 1468, 1430, 1370, 1336, 1263, 1198, 1166, 1136, 1096, 1003, 904, 867, 798, 728, 696, 633, 563, 500, 419; HRMS (ESI, m/z): $[M+H]^+$ calcd. for $C_{38}H_{49}FN_5O_{10}P$, 786.3274; found, 786.3295.

2'-(Bis-*O*-(4-heptanoyloxybenzyl))-(3'-deoxy-3'-fluoro-adenosine)-2',5'-diphosphate (15). In a nitrogen atmosphere, 0.18 g (0.24 mmol, 1.0 eq.) of the 2'-phosphate **14** was dissolved in 5 mL trimethyl phosphate (TMP). After the reaction solution was cooled to 0 °C, 0.10 mL (0.14 g, 0.94 mmol, 4.0 eq.) of phosphoryl chloride was added slowly and the reaction mixture was stirred at 0 °C for 6.5 h. To the reaction a 1 M TEAB buffer solution was added and the solvent was removed. The crude product was purified using automatic RP-18 chromatography (H_2O/CH_3CN gradient). Yield: 0.71 mg (0.073 mmol, 31 %) of a colorless solid. ^{1}H-NMR: (600 MHz, DMSO-*d*$_6$): *δ* 8.59 (s, 1H), 8.14 (s, 1H), 7.38 (s, 2H), 7.30-7.26 (m, 2H), 7.15-7.12 (m, 2H), 7.09-7.05 (m, 2H), 7.04-7.01 (m, 2H), 6.26 (d, *J* = 7.7 Hz, 1H), 5.81-5.72 (m, 1H) 5.43 (ddd, *J* = 53.5 Hz, *J* = 4.3 Hz, *J* = 4.3 Hz, 1H), 4.95 (dd, *J* = 11.9 Hz, *J* = 8.0 Hz, 1H), 4.87 (dd, *J* = 11.9 Hz, *J* = 8.5 Hz, 1H), 4.79-4.72 (m, 2H), 4.42 (ddd, *J* = 26.6 Hz, *J* = 4.5 Hz, *J* = 4.5 Hz, 1H), 4.04-3.92 (m, 2H), 2.98 (q, *J* = 7.3 Hz, 5H), 2.56 (dt, *J* = 7.5 Hz, *J* = 7.2 Hz, 4H), 1.66-1.60 (m, 4H), 1.38-1.33 (m, 4H), 1.32-1.26 (m, 8H), 1.16-1.11 (m, 9H), 0.91-0.84 (m, 6H); ^{13}C-NMR: (151 MHz, DMSO-*d*$_6$): *δ* 172.2, 156.6, 153.4, 150.9, 149.9, 139.9, 129.7, 129.6, 129.5 (d, *J* = 5.6 Hz), 122.4, 122.3, 122.3 (d, *J* = 6.6 Hz), 119.0, 91.5 (d, *J* = 188.4 Hz), 84.6, 82.2, 76.3, 69.1 (d, *J* = 4.8 Hz), 68.9 (d, *J* = 6.2 Hz), 63.5 (d, *J* = 11.2 Hz), 45.7, 33.9, 31.3, 28.5, 24.7, 22.4, 14.3, 8.92; ^{19}F-NMR: (565 MHz, DMSO-*d*$_6$): *δ* -196.92- -196.68 (m, 1F); ^{31}P-NMR: (243 MHz, DMSO-*d*$_6$): *δ* -2.27, -0.35; IR: (ATR) $\tilde{\nu}$ [cm^{-1}]: 2930, 2858, 1755, 1652, 1599, 1576, 1508, 1468, 1420, 1374, 1248, 1198, 1166, 1138, 1101, 1004, 917, 812, 799, 720, 684, 634, 562, 497, 396; HRMS (ESI, m/z): $[M-H]^+$ calcd. for $C_{38}H_{49}FN_5O_{13}P_2$, 866.2937; found, 866.2950. For Mass spectra (ESI$^+$) data see Supplementary Fig. 14.

2-(3-Bromophenyl)-4,5-dihydro-4,4-dimethyl-2-oxazole-2-yl (17). A catalytic amount of DMF was added to a suspension of 3.0 g (15 mmol, 1.0 eq.) 3-bromobenzoic acid **16** in 10 mL of thionyl chloride. The reaction mixture was stirred at 80 °C for 2 h, during which time the solid dissolved. The solution was concentrated in vacuo and coevaporated with diethyl ether. The solution of the residue dissolved in 20 mL dry CH_2Cl_2 was added slowly at 0 °C to 2.9 mL (30 mmol, 2.0 eq.) 2-amino-2-methyl-1-propanol dissolved in 20 mL dry CH_2Cl_2. The reaction mixture was stirred for 2 h at room temperature. At the end of the reaction the suspension was filtered and washed with CH_2Cl_2. The filtrate was concentrated in vacuo and the residue was dissolved in 10 mL of thionyl chloride. After the reaction was stirred at

room temperature for 16 h, the solution was concentrated in vacuo. The residue was dissolved in CH_2Cl_2 and neutralized with a 20 % NaOH-solution. The aqueous layer was extracted three times with diethyl ether. The combined organic layers were dried with Na_2SO_4 and the solvent was removed in vacuo. The crude product was purified by column chromatography (PE/EtOAc 9:1 v/v). Yield: 3.3 g (13 mmol, 87 %) as a colorless oil. ^{1}H-NMR: (500 MHz, $CDCl_3$): δ 8.12 (s, 1H), 7.87 (d, J = 7.2 Hz, 1H), 7.61 (d, J = 8.1 Hz, 1H), 7.28 (t, J = 8.2 Hz, 1H), 4.13 (s, 2H), 1.39 (s, 6H); ^{13}C-NMR: (125 MHz, $CDCl_3$): δ 161.4, 134.4, 131.4, 130.0, 127.0, 125.0, 122.6, 79.6, 79.4, 28.5; IR: (ATR) $\tilde{\nu}$ [cm^{-1}]: 3318, 2957, 2923, 1725, 1643, 1562, 1464, 1366, 1251, 1121, 1065, 970, 745, 713, 674; HRMS (ESI, m/z): $[M+H]^+$ calcd. for $C_{11}H_{13}BrNO$, 254.0175; found, 254.0301.

2,3,5-Tri-*O*-benzyl-1-(3-(4,5-dihydro-4,4-dimethyl-2-oxazole-2-yl)phenyl)-ribofuranose (18). In a nitrogen atmosphere, 0.50 g (2.0 mmol, 1.5 eq.) 2-(3-Bromophenyl)-4,5-dihydro-4,4-dimethyl-2-oxazole-2-yl **17** was dissolved in 3.0 mL dry THF and cooled to -78 °C. To the solution, 1.3 mL (2.1 mmol, 1.6 eq.) *n*-BuLi (1.6 M in hexane) was added slowly and stirred for 30 minutes at -78 °C. A solution of 0.54 g (1.3 mmol, 1.0 eq.) 2,3,5-tri-*O*-benzyl-ribono-γ-lactone in 3.0 mL dry THF was added dropwise and stirred at -78 °C for 1 h, while the reaction was stirred for another 2 h, it warmed up to -30 °C. The reaction was terminated by the addition of water. The aqueous layer was extracted three times with Et_2O, the combined organic layers were dried with Na_2SO_4 and the solvent was removed in vacuo. Under nitrogen atmosphere, the residue was dissolved in 3.0 mL dry CH_2Cl_2 and cooled to -78 °C. The addition of 0.62 mL (0.45 g, 3.9 mmol, 3.0 eq.) triethylsilane was followed by the slow addition of 0.41 mL (0.46 g, 3.3 mmol, 2.5 eq.) BF_3*Et_2O. The reaction solution was stirred for 16 h, slowly warming up from -78 °C to room temperature. Saturated $NaHCO_3$ solution was added, and the aqueous layer was extracted four times with CH_2Cl_2. The combined organic layers were dried over Na_2SO_4 and the solvent was removed in vacuo. The crude product was purified several times by column chromatography (PE/EtOAc 5:1 v/v), (toluene/EtOAc 8:1). Yield: 0.31 g (0.54 mmol, 41 %) as a colorless oil. ^{1}H-NMR: (400 MHz, DMSO-d_6): δ 7.95 (t, J = 1.8 Hz, 1H), 7.76 (dt, J = 7.7 Hz J = 1.5 Hz, 1H), 7.53 (dt, J = 7.9 Hz, J = 1.6 Hz, 1H), 7.39 (t, J = 7.7 Hz, 1H), 7.35-7.21 (m, 15H), 4.92 (d, J = 6.8 Hz, 1H), 4.62-4.43 (m, 6H), 4.27 (q, J = 3.9 Hz, 1H), 4.11-4.04 (m, 3H), 3.89 (dd, J = 6.9 Hz, J = 5.0 Hz, 1H), 3.69-3.59 (m, 2H), 1.27 (s, 6H); ^{13}C-NMR: (101 MHz, DMSO-d_6): δ 141.5, 138.8, 138.7, 138.4, 129.5, 129.4, 128.8, 128.7, 128.7, 128.6, 128.3, 128.1, 128.0, 128.0, 127.9, 127.5, 125.9, 84.0, 82.0, 81.7, 78.8, 77.7, 72.9, 71.7, 71.5, 70.7, 67.9, 28.7, 28.7; IR: (ATR) $\tilde{\nu}$ [cm^{-1}]:3063, 3030, 2965, 2892, 2865, 1649, 1604, 1586, 1496, 1454, 1353, 1314, 1251, 1194, 1081, 1060, 1027, 992, 970, 909, 805, 734, 718, 695, 645, 615, 571, 464, 422, 383; HRMS (ESI, m/z): $[M+H]^+$ calcd. for $C_{37}H_{40}NO_5$, 578.2901; found, 578.2902.

2,3,5-Tri-*O*-benzyl-ribofuranosyl-1-benzoic acid (19). In a nitrogen atmosphere, 2.0 g (3.5 mmol, 1.0 eq.) Compound **18** was dissolved in 10 mL nitromethane and 5 mL of methyl iodide and refluxed for 16 h. The solvent was removed in vacuo, the residue was solved in 30 mL CH_3OH and 30 mL of a KOH-solution (20 %). The suspension was refluxed for an additional 16 h and half the volume of the solution was removed in vacuo after completion of the reaction. The aqueous solution was then treated to a pH of 6 with 1 M HCl solution. The aqueous layer was extracted three times with EtOAc. The combined organic layers were dried with Na_2SO_4 and the solvent was removed in vacuo. The product was

used in the next reaction without further purification. Yield: 1.8 g (3.5 mmol, quantitatively) as a slightly yellowish oil. ^{1}H-NMR: (600 MHz, $CDCl_3$): δ 7.71 (s, 1H), 7.70 (s, 1H), 7.46 (d, J = 7.8 Hz, 1H), 7.31-7.10 (m, 16H), 5.00 (d, J = 6.9 Hz, 1H), 4.56-4.35 (m, 6H), 4.30-4.29 (m, 1H), 3.97-3.96 (m, 1H), 3.77-3.75 (m, 1H), 3.62 (dd, J = 10.6 Hz, J = 4.1 Hz, 1H), 3.55 (dd, J = 10.4 Hz, J = 3.7 Hz, 1H); ^{13}C-NMR: (151 MHz, $CDCl_3$): δ 167.6, 141.0, 138.1, 137.9, 137.6, 129.4, 128.9, 128.6, 128.5, 128.3, 128.2, 128.1, 127.8, 127.6, 127.4, 127.1, 124.3, 115.8, 83.9, 82.2, 82.1, 77.4, 73.5, 72.5, 72.1, 70.3; IR: (ATR) $\tilde{\nu}$ [cm^{-1}]: 3308, 2920, 2853, 1640, 1515, 1451, 1211, 1081, 908, 829, 731, 695; HRMS (ESI, m/z): [M+H]$^+$ calcd. for $C_{33}H_{31}O_6$, 523.2126; found, 523.2053.

Heptylchloroformate (21). In a nitrogen atmosphere, 17 g (57 mmol, 1.0 eq.) triphosgene was dissolved in 50 mL dry CH_2Cl_2 and cooled to 0 °C. To the solution, 20 g of 1-heptanol (0.17 mol, 3.0 eq.) was added dropwise. A solution of 13.9 mL dry pyridine (0.17 mol, 3.0 eq.) and 10 mL dry CH_2Cl_2 were added dropwise to the reaction mixture, meanwhile the temperature should be kept between 0-5 °C. The reaction mixture was stirred at room temperature for 15 h and then washed twice with cold H_2O. The organic layer was dried with Na_2SO_4, filtered and the solvent was removed in vacuo. The product was used without further purification. Yield: 28.7 g (161 mmol, 93 %) as a colorless oil. ^{1}H-NMR: (500 MHz, $CDCl_3$): δ 4.31 (t, J = 6.7 Hz, 2H), 1.77-1.67 (m, 2H), 1.48-1.18 (m, 8H), 0.92-0.86 (m, 3H); ^{13}C-NMR: (126 MHz, $CDCl_3$): δ 151.1, 72.8, 32.0, 29.1, 28.7, 25.9, 22.9, 14.4; HRMS (ESI, m/z): [M+Na]$^+$ calcd. for $C_8H_{15}ClO_2$, 200.0580; found, 200.1874.

4-(Hydroxymethyl)phenylheptyl carbonate (22). General procedure A with 7.5 g (60 mmol, 1.1 eq.) 4-hydroxybenzyl alcohol, 5.4 g (54 mmol, 1.0 eq.) dry Et_3N dissolved in 40 mL dry CH_2Cl_2 and dropwise addition of 9.7 g (54 mmol, 1.0 eq.) heptylchloroformate **21** dissolved in 20 mL dry CH_2Cl_2 at 0 °C. The reaction was stirred at room temperature. The crude product was purified by column chromatography on silica gel (PE/EtOAc 3:2 v/v). Yield: 10.6 g (39.5 mmol, 74 %) as a colorless oil. ^{1}H-NMR: (400 MHz, $CDCl_3$): δ 7.44-7.32 (m, 2H), 7.19-7.13 (m, 2H), 4.68 (s, 2H), 4.24 (t, J = 6.7 Hz, 2H), 1.73 (dt, J = 8.2 Hz, J = 6.6 Hz, 2H), 1.46-1.26 (m, 8H), 0.94-0.85 (m, 3H); ^{13}C-NMR: (101 MHz, $CDCl_3$): δ 153.9, 150.7, 138.8, 128.2, 121.3, 69.2, 64.8, 31.8, 29.0, 28.7, 25.8, 22.7, 13.7; IR: (ATR) $\tilde{\nu}$ [cm^{-1}]: 2956, 2857, 1758, 1509, 1391, 1209, 1048, 1012, 824, 726, 604, 507; HRMS (ESI, m/z): [M+Na]$^+$ calcd. for $C_{15}H_{22}O_4$, 289.1402; found, 289.1342.

1-(4-(((Heptyloxy)carbonyl)oxy)-benzyl)benzoyl-2,3,5-tri-*O*-benzyl-ribofuranose (23). In a nitrogen atmosphere, 0.50 g (0.96 mmol, 1.0 eq.) 2,3,5-tri-*O*-benzyl-ribofuranosyl-1-benzoic acid **19**, 0.17 mL (0.26 g, 1.1 mmol, 1.1 eq.) 2,4,6-trichlorobenzoyl chloride and 0.16 mL (0.12 g, 1.2 mmol, 1.2 eq.) Et_3N were dissolved in 10 mL dry THF. This solution was stirred for 30 minutes at room temperature. A solution of 0.31 g (1.2 mmol, 1.2 eq.) 4-(hydroxymethyl)phenylheptylcarbonate **22**, and 40 mg (0.29 mmol, 0.3 eq.) DMAP in 6 mL THF was added to the reaction mixture. The reaction was stirred for 3 h at 70 °C and for 16 h at room temperature. After completion of the reaction, saturated NaCl solution was added. The aqueous layer was extracted three times with CH_2Cl_2. The combined organic layers were dried with Na_2SO_4 and concentrated in vacuo. The crude product was purified by column chromatography on silica gel (PE/EtOAc 6:1 v/v). Yield: 0.43 g (0.56 mmol, 58 %) as a colorless oil. ^{1}H-NMR: (400 MHz, DMSO-

d_6): δ 8.05 (m, 1H), 7.91 (dt, J = 7.7 Hz, J = 1.5 Hz, 1H), 7.66 (dt, J = 7.8 Hz, J = 1.5 Hz, 1H), 7.51-7.47 (m, 2H), 7.47-7.44 (m, 1H), 7.35-7.27 (m, 10H), 7.25-7.17 (m, 7H), 5.33 (m, 2H), 4.95 (d, J = 6.9 Hz, 1H), 4.61-4.42 (m, 6H), 4.28 (dt, J = 4.0 Hz, J = 3.8 Hz, 1H), 4.19 (t, J = 6.6 Hz, 2H), 4.08 (dd, J = 5.0 Hz, J = 3.5 Hz, 1H), 3.90 (dd, J = 7.0 Hz, J = 4.9 Hz, 1H), 3.68-3.57 (m, 2H), 1.66 (quin., J = 6.7 Hz, 2H), 1.93-1.21 (m, 8H), 0.90-0.83 (m, 3H); ^{13}C-NMR: (101 MHz, DMSO-d_6): δ 166.0, 153.6, 150.9, 141.9, 138.7, 138.6, 138.4, 134.5, 131.6, 130.0, 129.8, 129.2, 129.0, 128.7, 128.7, 128.6, 128.3, 128.0, 128.0, 127.9, 127.8, 127.3, 121.84, 84.0, 82.0, 81.5, 77.7, 72.9, 71.6, 71.5, 70.7, 69.0, 66.0, 31.5, 28.7, 28.5, 25.5, 22.5, 14.4; IR: (ATR) $\tilde{\nu}$ [cm^{-1}]: 3066, 2924, 2857, 1719, 1607, 1495, 1373, 1252, 1200, 1119, 1079, 843, 734, 696, 536; HRMS (ESI, m/z): [M+Na]$^+$ calcd. for $C_{48}H_{52}O_9$, 795.3504; found, 795.3510.

(1-(4-(((Heptyloxy)carbonyl)oxy)benzyl)benzoyl)ribofuranose (24). In a nitrogen atmosphere, 0.38 g (0.49 mmol, 1.0 eq.) 2,3,5-tri-*O*-benzyl-ribofuranosyl-1-(4- (((heptyloxy)carbonyl)oxy)benzyl)benzoate **23** was dissolved in 10 mL dry CH_2Cl_2 and cooled to -78 °C. To this mixture, 1.7 mL (1.7 mmol, 3.5 eq.) boron trichloride solution (1 M in CH_2Cl_2) was slowly added within 15 minutes. The reaction was stirred for 2 h, slowly getting warmer. The mixture was added to a 0 °C cooled saturated $NaHCO_3$ solution was added and the aqueous layer was extracted three times with CH_2Cl_2. The combined organic layers were dried with Na_2SO_4 and the solvent was removed in vacuo. The crude product was purified by column chromatography on silica gel (CH_2Cl_2/CH_3OH 20:1 v/v → CH_2Cl_2/CH_3OH 15:1 v/v). Yield: 0.16 g (0.33 mol, 67 %) as a colorless oil. ^{1}H-NMR: (600 MHz, DMSO-d_6): δ 8.02 (m, 1H), 7.91 (dt, J = 7.7 Hz, J = 1.5 Hz, 1H), 7.72 (dt, J = 7.8 Hz, J = 1.7 Hz, 1H), 7.55-7.52 (m, 2H), 7.51 (t, J = 7.7 Hz, 1H), 7.29-7.25 (m, 2H), 5.37 (s, 2H), 5.06 (d, J = 7.1 Hz, 1H), 4.96 (d, J = 4.8 Hz, 1H), 4.84 (d, J = 5.5 Hz, 1H), 4.64 (d, J = 7.4 Hz, 1H), 4.20 (t, J = 6.6 Hz, 2H), 3.92-3.39 (m, 1H), 3.86-3.84 (m, 1H), 3.70-3.65 (m, 1H), 3.60-3.53 (m, 2H), 1.70-1.64 (m, 2H), 1.38-1.22 (m, 8H), 0.89-0.85 (m, 3H); ^{13}C-NMR: (151 MHz, DMSO-d_6): δ 166.0, 153.4, 150.8, 142.8, 134.5, 131.6, 129.8, 129.1, 128.7, 127.4, 121.9, 85.9, 82.7, 78.1, 71.9, 69.1, 66.0, 62.5, 31.6, 28.7, 28.5, 25.6, 22.5, 14.4; IR: (ATR) $\tilde{\nu}$ [cm^{-1}]: 3362, 2955, 2858, 1761, 1716, 1447, 1254, 1193, 1103, 1025, 778; HRMS (ESI, m/z): [M+Na]$^+$ calcd. for $C_{27}H_{34}O_9$, 525.2095; found, 525.2091.

((Ribofuranosyl)-1-(4-(((heptyloxy)carbonyl)oxy)benzyl-benzoyl)-5-phosphate (25). General procedure C with 70.0 mg (297 µmol, 1.00 eq.) (1-(4-(((heptyloxy)carbonyl)oxy)benzyl)benzoyl)ribofuranose **24**, 25.0 µL (274 µmol, 2.00 eq.) $P(O)Cl_3$, 2 mL dry trimethyl phosphate and 83.0 µL (349 µL, 2.50 eq.) tri-*n*-butylamine. The residue was purified using automatic RP-18 chromatography (H_2O/CH_3CN gradient) and finally lyophilized. Yield: 39.5 mg (233 µmol, 42 %) as a colorless solid. The yield was calculated with one triethylammonium counterion. ^{1}H-NMR: (400 MHz, CH_3OD): δ 8.11 (s, 1H), 7.95 (d, J = 7.8 Hz, 1H), 7.72 (d, J = 7.7 Hz, 1H), 7.50-7.48 (m, 2H), 7.45 (t, J = 8.2 Hz, 1H), 7.20-7.18 (m, 2H), 5.35 (s, 2H), 4.73 (d, J = 7.0 Hz, 1H), 4.21 (t, J = 6.6 Hz, 2H), 4.03-3.99 (m, 1H), 3.99-3.96 (m, 1H), 3.83-3.79 (m, 1H), 3.77 (dd, J = 11.9 Hz, J = 3.9 Hz, 1H), 3.71 (dd, J = 11.9 Hz, J = 4.8 Hz, 1H), 3.13 (q, J = 7.4 Hz, 6H), 1.70 (quin., J = 6.4 Hz, 2H), 1.42-1.26 (m, 8H), 1.27 (t, J = 7.5 Hz, 9H), 0.89 (t, J = 6.8 Hz, 3H); ^{13}C-NMR: (151 MHz, CH_3OD): δ 165.4, 155.2, 152.3, 141.5, 135.4, 132.1, 131.5, 130.6, 130.2, 129.9, 128.2, 122.4, 85.5, 84.8, 84.2, 79.5, 70.0, 67.1, 65.6 (d, J = 5.4 Hz), 47.7, 32.9, 30.0, 29.7, 26.8, 23.6, 14.4, 9.2; ^{31}P-NMR: (162 MHz, CH_3OD): δ 0.73; IR: (ATR) $\tilde{\nu}$ [cm^{-1}]: 3251, 2928,

2857, 2480, 1759, 1717, 1453, 1247, 1217, 1195, 1046, 924, 834, 781, 505; HRMS (ESI, m/z): $[M-H]^-$ calcd. for $C_{27}H_{34}O_{12}P$, 581.1793; found, 581.1797. For Mass spectra (ESI^+) data see Supplementary Fig. 15.

MASTER-NAADP (26). General procedure D with 23.8 µL (171 µmol, 10.0 eq.) TFAA, 37.9 µL (274 µmol, 16.0 eq.) Et_3N and 11.7 mg (17.1 µmol, 1.00 eq.) of the phosphate **25**, 8.20 µL (103 µmol, 6.00 eq.) N-methylimidazole (NMI), 23.7 µL (171 µmol, 10.0 eq.) Et_3N and 18.0 mg (18.6 µmol, 1.10 eq.) phosphate **15** were used. The crude product was purified using automatic RP-18 chromatography (H_2O/CH_3CN gradient) and finally lyophilized. Yield: 16.0 mg (9.80 µmol, 57 %) as a colorless solid. The yield was calculated with two triethylammonium counterions. ^1H-NMR: (600 MHz, CH_3OD): δ 8.67 (s, 1H), 8.16 (s, 1H), 8.06 (s, 1H), 7.91 (d, J = 8.0 Hz, 1H), 7.80 (d, J = 7.8 Hz, 1H), 7.52-7.51 (m, 2H), 7.44 (t, J = 7.7 Hz, 1H), 7.21-7.18 (m, 4H), 7.15-7.14 (m, 2H), 7.01-6.98 (m, 4H), 6.34 (d, J = 7.5 Hz, 1H), 5.71-5.65 (m, 1H), 5.56-5.46 (m, 1H), 5.36 (s, 2H), 4.84-4.81 (m, 4H), 4.76 (d, J = 7.01 Hz, 1H), 4.62-4.58 (m, 2H), 4.36-4.27 (m, 6H), 4.23 (t, J = 6.7 Hz, 2H), 4.19-4.17 (m, 2H), 4.11 (q, J = 7.2 Hz, 6H), 3.96-3.94 (m, 1H), 3.19 (q, J = 7.2 Hz, 6H), 2.57 (t, J = 7.5 Hz, 4H), 1.75-1.70 (m, 6H), 1.46-1.34 (m, 20H), 1.30 (t, J = 7.3 Hz, 9H), 1.25 (t, J = 7.2 Hz, , 9H), 0.95-0.91 (m, 9H); ^{31}P-NMR: (162 MHz, CH_3OD): δ -2.89, -10.99 (d, J = 21.8 Hz), -11.64 (d, J = 21.8 Hz); ^{19}F-NMR: (565 MHz, CH_3OD): δ -198.30; HRMS (ESI, m/z): $[M-H]^-$ calcd. for $C_{65}H_{82}FN_5O_{24}P_3$, 1428.4552; found, 1428.4551. For Mass spectra (ESI^+) data see Supplementary Fig. 16.

***tert*-Butyl-(4-(((heptyloxy)carbonyl)oxy)benzyl)carbamate (28).** In a nitrogen atmosphere, 1.4 mL (6.4 mmol, 1.1 eq.) Boc_2O was slowly added to a solution of 0.75 mg (6.1 mmol, 1.0 eq.) 4-hydroxybenzylamine **27** in 5 mL dry DMF and dry pyridine (5:1 v/v) which was cooled to 0 °C. After the reaction mixture was stirred for 3 h at room temperature 30 mL H_2O and 30 mL EtOAc were added. The organic layer was washed once with saturated NaCl solution, dried over Na_2SO_4, filtered and the solvent was removed in vacuo. The next step starting from the residue was carried out analog to general procedure A. A solution of 0.84 mL (6.1 mmol, 1.0 eq.) dry Et_3N in 40 mL dry CH_2Cl_2 was added to the crude product followed by addition of 0.98 g (5.5 mmol, 0.90 eq.) heptylchloroformate **21** dissolved in 20 mL dry CH_2Cl_2. The crude product was purified by column chromatography on silica gel (PE/EtOAc 4:1 v/v). Yield: 1.3 g (3.5 mmol, 57 %) as a colorless oil. ^1H-NMR: (400 MHz, $CDCl_3$): δ 7.30-7.28 (m, 2H), 7.14-7.12 (m, 2H), 4.84 (bs, 1H), 4.31 (s, 2H), 4.24 (t, J = 6.8 Hz, 2H), 1.74 (dt, J = 7.2 Hz, J = 6.9 Hz, 2H), 1.45 (s, 9H), 1.42-1.27 (m, 8H), 0.89 (t, J = 7.1 Hz, 3H); ^{13}C-NMR: (101 MHz, $CDCl_3$): δ 53.9, 150.5, 136.9, 127.8, 121.4, 79.8, 69.2, 44.2, 31.8, 29.0, 28.7, 28.5, 25.8, 21.7, 14.2; IR: (ATR) $\tilde{\nu}$ [cm^{-1}]: 3361, 2957, 2927, 2857, 1760, 1697, 1507, 1390, 1245, 1216, 1162, 1073, 780, 730; HRMS (ESI, m/z): $[M+Na]^+$ calcd. for $C_{20}H_{31}NO_5$, 388.2100; found, 388.2156.

4-(Aminomethyl)phenylheptylcarbonate*hydrochloride (29). In a nitrogen atmosphere, 1.3 g (3.4 mmol, 1.0 eq.) *tert*-butyl(4-(((heptyloxy)carbonyl)oxy)benzyl)carbamate **28** was dissolved in 20 mL dry EtOH and cooled to 0 °C. After the slow addition of 4.9 mL (68 mmol, 20 eq.) acetyl chloride the mixture was stirred for 3 h at room temperature. The solvent was removed in vacuo and the product

was used without further purification. Yield: 0.93 g (3.1 mmol, 90 %) as a slightly beige solid. ^{1}H-NMR: (500 MHz, $CDCl_3$): δ 7.54-7.52 (m, 2H), 7.30-7.28 (m, 2H), 4.26 (t, J = 6.6 Hz, 2H), 4.16 (s, 2H), 1.75 (dt, J = 7.3 Hz, J = 6.6 Hz, 2H), 1.47-1.33 (m, 8H), 0.94 (t, J = 7.1 Hz, 3H); ^{13}C-NMR: (125 MHz, $CDCl_3$): δ 155.1, 153.3, 132.2, 131.5, 123.1, 70.2, 43.7, 32.9, 30.0, 29.7, 26.8, 23.6, 14.4; IR: (ATR) $\tilde{\nu}$ [cm^{-1}]: 3394, 2927, 2511, 1756, 1513, 1462, 1256, 1219, 825; HRMS (ESI, m/z): [M+Na]$^+$ calcd. for $C_{15}H_{14}NO_3$, 266.1756; found, 266.1691.

2,3,5-Tri-*O*-benzyl-ribofuranosyl-1-(4-(((heptyloxy)carbonyl)oxy)benzyl)benzamide (30).
In a nitrogen atmosphere, 0.64 g (1.2 mmol, 1.0 eq.) 2,3,5-tri-*O*-benzyl-D-ribofuranosyl-1-benzoic acid **19** was dissolved in 9 mL dry CH_2Cl_2. To this solution 0.37 mL (2.7 mmol, 2.2 eq.) Et_3N and 0.56 g (1.5 mmol, 1.2 eq.) (2-(1*H*-benzotriazol-1-yl)-1,1,3,3-tetramethyluronium-hexafluorophosphate (HBTU) were added. After the reaction mixture stirred for 30 min at room temperature 0.55 g (1.8 mmol, 1.5 eq.) of 4-(aminomethyl)phenylheptylcarbonate **29** and 45 mg (0.37 mmol, 0.30 eq.) DMAP were added. The solution was stirred for 24 h at room temperature and terminated with aqueous saturated sodium chloride solution. The aqueous phase was extracted with CH_2Cl_2 and the combined organic phases were dried over sodium sulfate and purified by column chromatography on silica gel (PE/EA 2:1 v/v).
Yield: 0.45 g (0.58 mmol, 48 %) as a yellowish oil. ^{1}H-NMR: (500 MHz, DMSO-d_6): δ 9.05 (t, J = 6.0 Hz, 1H), 7.93 (m, 1H), 7.80 (dt, J = 7.9 Hz, 1.5 Hz, 1H), 7.55 (d, J = 7.7 Hz, 1H), 7.40 (t, J = 7.7 Hz, 1H), 7.37-7.19 (m, 17H), 7.16 (d, J = 8.6 Hz, 2H), 4.93 (d, J = 6.8 Hz, 1H), 4.62-4.51 (m, 6H), 4.49-4.44 (m, 2H), 4.27-4.25 (m, 1H), 4.18 (t, J = 6.6 Hz, 2H), 4.11-4.06 (m, 1H), 3.93 (dd, J = 6.8 Hz, 4.9 Hz, 1H), 3.67 (dd, J = 10.6 Hz, J = 4.1 Hz, 1H), 3.63 (dd, J = 10.6 Hz, J = 4.5 Hz, 1H), 1.65 (quin., J = 6.8 Hz, 2H), 1.39-1.21 (m, 8H), 0.88 (t, J = 6.7 Hz, 3H); ^{13}C-NMR: (125 MHz, DMSO-d_6): δ 166.1, 153.1, 149.6, 140.8, 138.3, 138.2, 137.9, 137.6, 134.3, 129.0, 128.4, 128.3, 128.2, 128.1, 127.8, 127.5, 127.4, 126.5, 125.4, 121.1, 83.4, 81.5, 81.3, 77.3, 72.4, 71.2, 70.9, 70.3, 68.5, 42.0, 31.1, 28.2, 27.9, 25.1, 21.9, 13.9; IR: (ATR) $\tilde{\nu}$ [cm^{-1}]: 3327, 2925, 2857, 1757, 1245, 1125, 1026, 816, 733, 695; HRMS (ESI, m/z): [M+H]$^+$ calcd. for $C_{48}H_{53}NO_8$, 772.3844; found, 772.3885.

(1-(4-(((Heptyloxy)carbonyl)oxy)benzyl)benzamid)-ribofuranose (31). In a nitrogen atmosphere, 270 mg (350 µmol, 1.00 eq.) 2,3,5-tri-*O*-benzyl-ribofuranosyl-1-(4-(((heptyloxy)-carbonyl)oxy)benzyl)benzamide **30** was dissolved in 10 mL dry CH_2Cl_2 and cooled to -78 °C. To the solution, 1.85 mL (1.85 mmol, 5.29 eq.) boron trichloride solution (1 M in CH_2Cl_2) were slowly added within 15 minutes. The solution was warmed to -10 °C and stirred for 30 minutes. A saturated $NaHCO_3$ solution was added and the aqueous layer was extracted three times with CH_2Cl_2. The combined organic layers were dried with Na_2SO_4 and the solvent was removed in vacuo. The crude product was purified by column chromatography on silica gel (CH_2Cl_2/CH_3OH 9:1 v/v). Yield: 145 mg (291 µmol, 83 %) as a colorless oil. ^{1}H-NMR: (600 MHz, $CDCl_3$): δ 7.80 (s, 1H), 7.74-7.68 (m, 1H), 7.56 (d, J = 7.3 Hz, 1H), 7.40 (d, J = 7.0 Hz, 1H), 7.24-7.22 (m, 2H), 7.03-7.02 (m, 2H), 6.02-5.91 (m, 1H), 4.70-4.66 (m, 1H), 4.47-4.42 (m, 2H), 4.18 (t, J = 6.9 Hz, 2H), 4.05-4.01 (m, 1H), 3.98-3.94 (m, 1H), 3.85-3.81 (m, 1H), 3.77-3.72 (m, 1H), 3.67-3.62 (m, 1H), 1.70 (quin., J = 7.5 Hz, 2H), 1.40-1.25 (m, 8H), 0.88 (t, J = 6.7 Hz, 3H); ^{13}C-NMR: (101 MHz, $CDCl_3$): δ 167.2, 154.2, 150.6, 139.6, 135.7, 134.0, 132.0, 129.0, 128.7, 127.0, 125.9, 121.4, 84.9,

83.8, 76.8, 74.0, 69.0, 62.8, 46.5, 31.8, 29.0, 28.2, 25.8, 22.7, 14.2; HRMS (ESI, m/z): $[M+Na]^+$ calcd. for $C_{27}H_{36}NO_8$, 502.2441; found, 502.2414.

[(Ribofuranosyl)-1-(4-(((heptyloxy)carbonyl)oxy)benzyl-benzamide]-5-phosphate (32). General procedure C with 20.0 mg (39.9 µmol, 1.00 eq.) (1-(4-(((heptyloxy)carbonyl)oxy)benzyl)benzamide)-ribofuranose **31** and 146 µL (1.60 mmol, 40.0 eq.) $P(O)Cl_3$ in 2 mL dry trimethyl phosphate. The residue was purified using automatic RP-18 chromatography (H_2O/CH_3CN gradient) and finally lyophilized. Yield: 27.0 mg (39.5 µmol, quantitativly) as a colorless solid. The yield was calculated with one triethylammonium counterion. ^{1}H-NMR: (600 MHz, CH_3OD): δ 8.06 (s, 1H), 7.78 (d, J = 7.7 Hz, 1H), 7.63 (d, J = 7.7 Hz, 1H), 7.45-7.42 (m, 3H), 7.13-7.11 (m, 2H), 4.76 (d, J = 7.6 Hz, 1H), 4.58 (s, 2H), 4.21-4.20 (m, 3H), 4.14-4.12 (m, 1H), 4.11-4.08 (m, 1H), 4.08-4.05 (m, 1H), 4.01-3.99 (m, 1H), 3.05 (q, J = 7.4 Hz, 6H), 1.71 (quin., J = 6.7 Hz, 2H), 1.43-1.30 (m, 8H), 1.23 (t, J = 7.5 Hz, 9H), 0.91 (t, J = 7.0 Hz, 3H); ^{13}C-NMR: (151 MHz, CH_3OD): δ 170.0, 155.3, 151.7, 142.9, 138.4, 135.5, 131.1, 129.8, 129.5, 128.0, 126.0, 122.3, 85.6 (d, $^3J_{CP}$ = 8.7 Hz), 84.7, 79.3, 73.6, 69.9, 66.4 (d, $^2J_{CP}$ = 5.4 Hz), 47.5, 44.0, 32.9, 30.0, 29.7, 26.8, 23.6, 14.4, 9.4; ^{31}P-NMR: δ [ppm] (162 MHz, CH_3OD): 1.11; HRMS (ESI, m/z): $[M-H]^-$ calcd. for $C_{27}H_{35}NO_{11}P$, 580.1953; found, 580.1931.

4-(Hydroxymethyl)phenylhexanoate (33). General procedure A with 4.0 g (32 mmol, 1.1 eq.) 4-hydroxybenzyl alcohol **10**, 4.1 mL (29 mmol, 1.0 eq.) Et_3N dissolved in 40 mL dry CH_2Cl_2. A solution of 4.1 mL (29 mmol, 1.0 eq.) hexanoic acid chloride in 20 mL dry CH_2Cl_2 was added. The reaction was stirred at room temperature. The crude product was purified by column chromatography on silica gel (PE/EtOAc 2:1 v/v). Yield: 2.89 g (13.0 mmol, 44 %) as a colorless oil. ^{1}H-NMR: (500 MHz, $CDCl_3$): δ 7.35 (m, 2H), 7.06 (m, 2H), 4.66 (s, 2H), 2.55 (t, J = 7.5 Hz, 2H), 1.76 (quint., J = 7.5 Hz, 2H), 1.41-1.36 (m, 4H), 0.93 (t, J = 6.8 Hz, 3H); ^{13}C-NMR: (126 MHz, $CDCl_3$): δ 173.1, 150.3, 139.6, 128.2, 121.8, 66.5, 34.5, 32.4, 26.0, 22.4, 13.7; IR: (ATR) $\tilde{\nu}$ [cm^{-1}]: 2956, 2867, 1736, 1609, 1463, 1198, 1161, 1144, 1038, 892, 823, 505; HRMS (ESI, m/z): $[M+Na]^+$ calcd. for $C_{13}H_{19}O_3$, 223.1329; found, 223.1231.

Di*iso*propyl-*N*-amino(bis-4-(hydroxymethyl)phenylhexanoate)phosphoramidite (34). General procedure B with 310 µL (1.69 mmol, 1.00 eq.) dichloro-*N,N*-di*iso*propylaminophosphoramidite, 750 mg (3.37 mmol, 2.00 eq.) 4-(hydroxymethyl)phenylhexanoate **33** and 560 µL (4.05 mmol, 2.20 eq.) Et_3N. The crude product was purified by column chromatography on silica gel (PE/EtOAc/Et_3N 97:2:1 v/v/v). Yield: 701 mg (1.22 mmol, 72 %) as a colorless viscous liquid. ^{1}H-NMR: (400 MHz, $CDCl_3$): δ 7.36-7.34 (m, 4H), 7.04-7.02 (m, 4H), 4.80-4-64 (m, 4H), 3.73-3.64 (m, 2H), 2.55 (t, J = 7.6 Hz, 2H), 1.76 (quint., J = 7.4 Hz, 2H), 1.43-1.34 (m, 4H), 1.20 (d, J = 6.7 Hz, 12H), 0.93 (t, J = 7.1 Hz, 3H); ^{13}C-NMR: (101 MHz, $CDCl_3$): δ 172.5, 150.0, 137.2, 127.8, 121.5, 65.1, 64.9, 43.8, 43.2, 36.0, 31.4, 24.8, 22.5, 14.1; ^{31}P-NMR: (162 MHz, $CDCl_3$): δ 147.88; IR: (ATR) $\tilde{\nu}$ [cm^{-1}]: 2921, 2852, 2388, 1707, 1612, 1467, 1393, 1139, 976, 823, 546; HRMS (ESI, m/z): $[M+K]^+$ calcd. for $C_{32}H_{48}NO_6P$, 612.2856; found, 612.2546.

3',5'-*O*-(1,1,3,3-Tetraisopropyldisiloxan-1,3-diyl)-adenosine (36). In a nitrogen atmosphere, 3.0 g (11 mmol, 1.0 eq.) adenosine **35** was dissolved in 40 mL dry pyridine. To the solution 0.70 mg (5.6 mmol, 0.50 eq.) DMAP was added and 4.0 mL (13 mmol, 1.2 eq.) $TIPDSiCl_2$ was added dropwise.

The reaction solution was stirred for 16 h at room temperature. After completion of the reaction, the solvent was removed in vacuo. The crude product was purified by column chromatography on silica gel (CH_2Cl_2/CH_3OH 19:1 v/v). Yield: 5.2 g (10 mmol, 91 %) as a colorless solid. ^{1}H-NMR: (500 MHz, $CDCl_3$): *δ* 8.29 (s, 1H), 7.99 (s, 1H), 5.98 (d, *J* = 1.4 Hz, 1H), 5.95 (s, 2H), 5.09 (dd, *J* = 7.6 Hz, *J* = 5.4 Hz, 1H), 4.58 (dd, *J* = 5.5 Hz, *J* = 1.4 Hz, 1H), 4.19–4.01 (m, 3H), 3.25 (s, 1H), 1.16–1.00 (m, 28H); ^{13}C-NMR: (125 MHz, $CDCl_3$): *δ* 155.0, 151.8, 149.2, 140.2, 120.5, 89.8, 82.4, 75.3, 70.9, 61.9, 17.6, 17.5, 17.5, 17.4, 17.3, 17.2, 17.1, 17.1, 13.4, 13.0, 12.9, 12.8; IR: (ATR) $\tilde{\nu}$ [cm^{-1}]: 3320, 3128, 2866, 1645, 1576, 1465, 1383, 1293, 1156, 1034, 904, 856, 770, 598, 451; HRMS (ESI, m/z): $[M+H]^+$ calcd. for $C_{22}H_{40}N_5O_5Si_2$, 510.2562; found, 510.2564.

3‘,5‘-*O*-(1,1,3,3-Tetra*iso*propyldisiloxan-1,3-diyl)-adenosine-2’-bis(4-(hydroxymethyl)-phenylhex-anoate)-2‘-phosphate (37). In a nitrogen atmosphere, 0.57 g (1.1 mmol, 1.0 eq.) 3',5'-*O*-(1,1,3,3-tetra*i-so*propyl-disiloxan-1,3-diyl)-adenosine **36** was dissolved in dry CH_2Cl_2, the solution was cooled to 0 °C and 0.70 g (1.2 mmol, 1.1 eq.) protected amidite **34** was added. To this mixture 1.0 mL (1.7 mmol, 1.5 eq.) DCI (0.25 M in CH_3CN) was added dropwise. After stirring for 2 h at room temperature 54 µL (1.3 mmol, 2.0 eq.) *tert*-BuOOH (5.5 M in *n*-decane) was added. The reaction mixture was stirred for 1 h and the volatile components were removed under reduced pressure. The crude product was purified by column chromatography on silica gel (CH_2Cl_2/CH_3OH 19:1 v/v). Yield: 780 mg (781 µmol, 70 %) as a colorless viscous liquid. ^{1}H-NMR: (500 MHz, $CDCl_3$): *δ* 8.21 (s, 1H), 7.95 (s, 1H), 7.37-7.35 (m, 2H), 7.32-7.29 (m, 2H), 7.09-7.03 (m, 4H), 6.09 (bs, 2H), 6.01 (s, 1H), 5.30 (s, 1H), 5.13-5.02 (m, 4H), 4.99 (ddd, *J* = 9.2 Hz, *J* = 4.8 Hz, *J* = 1.9 Hz, 1H), 4.18 (dd, *J* = 13.3 Hz, *J* = 1.8 Hz, 1H), 4.07 (dt, *J* = 9.3 Hz, *J* = 2.1 Hz, 1H), 4.01 (dd, *J* = 13.2 Hz, *J* = 2.6 Hz, 1H), 2.57-2.53 (m, 4H), 1.79-1.73 (m, 4H), 1.43-1.35 (m, 8H), 1.12-1.00 (m, 28H), 0.93 (t, *J* = 6.4 Hz, 6H); ^{13}C-NMR: (125 MHz, $CDCl_3$): *δ* 172.3, 154.7, 151.7, 149.0, 145.4, 140.0, 133.0, 129.4, 129.3, 122.1, 122.0, 120.2, 88.7 (d, *J* = 4.4 Hz), 81.6, 79.9 (d, *J* = 5.6 Hz), 69.2 (d, *J* = 5.2 Hz), 68.5 (d, *J* = 4.7 Hz), 60.0, 34.5, 31.4, 25.9, 22.5, 17.6, 17.4, 17.2, 17.1, 17.0, 17.1, 17.0, 14.1, 13.5, 13.1, 13.0, 12.7; ^{31}P-NMR: (162 MHz, $CDCl_3$): *δ* -1.45; IR: (ATR) $\tilde{\nu}$ [cm^{-1}]: 3109, 2929, 2865, 1758, 1687, 1508, 1463, 1203, 1141, 1036, 882, 690; HRMS (ESI, m/z): $[M+H]^+$ calcd. for $C_{48}H_{73}N_5O_{12}PSi_2$, 998.4532; found, 998.3638.

3‘-*O*-(1,1,3,3-Tetra*iso*propyldisiloxan-1,3-diyl)-adenosine-2’-bis(4-(hydroxymethyl)phenylhexa-noate)-2‘-phosphate (38). A solution of 0.75 g (0.75 mmol, 1.0 eq.) 3',5'-*O*-(1,1,3,3-tetrai-sopropyldisiloxan-1,3-diyl)-adenosine-2'-bis(4-(hydroxymethyl)phenylhexanoyl)phosphate **37** in THF was cooled to 0 °C. A mixture of water, THF and TFA (THF/H_2O/TFA 4:1:1 v/v/v) was added slowly. The reaction mixture was kept at 0 °C and stirred for 3 h. After adding saturated $NaHCO_3$ solution, the aque-ous layer was extracted with EtOAc. The combined organic layers were dried over Na_2SO_4 and the solvent was removed. The crude product was purified by column chromatography on silica gel (CH_2Cl_2/CH_3OH 19:1 v/v). Yield: 0.63 mg (0.62 µmol, 83 %) as a colorless oil. ^{1}H-NMR: (500 MHz, $CDCl_3$): *δ* 8.18 (s, 1H), 7.76 (s, 1H), 7.15-7.11 (m, 4H), 7.03-6.97 (m, 4H), 6.12 (bs, 2H), 6.00 (d, *J* = 6.8 Hz, 1H,), 5.55-5.51 (m, 1H), 4.93 (dd, *J* = 4.7 Hz, *J* = 1.4 Hz, 1H), 4.81-4.64 (m, 4H), 4.27-4.26 (m, 1H), 3.92 (dd, 1H, *J* = 13.0 Hz, *J* = 1.6 Hz), 3.76-3.73 (m, 1H), 2.53 (t, 4H, *J* = 7.6 Hz), 1.77-1.71 (m, 4H), 1.41-1.33 (m, 8H), 1.09-1.05 (m, 28H), 0.92 (t, *J* = 6.5 Hz, 6H); ^{13}C-NMR: (125 MHz, $CDCl_3$): *δ*

172.3, 155.9, 152.4, 151.1, 148.7, 140.9, 132.4, 129.2, 129.1, 122.0, 119.8, 88.8 (d, *J* = 6.4 Hz), 78.4, 77.4 (d, *J* = 5.2 Hz), 71.6 (d, *J* = 4.6 Hz), 69.4 (t, *J* = 5.4 Hz), 62.4, 34.9, 31.4, 24.7, 22.4, 17.6, 17.5, 17.4, 17.3, 14.0, 13.7, 13.6, 13.5; ^{31}P-NMR: (162 MHz, $CDCl_3$): *δ* -1.74; IR: (ATR) $\tilde{\nu}$ [cm^{-1}]: 3190, 2932, 2864, 1757, 1575, 1509, 1203, 1145, 1052, 884, 687; HRMS (ESI, m/z): [M+H]$^+$ calcd. for $C_{48}H_{75}N_5O_{13}PSi_2$, 1016.4638; found, 1016.4457.

5'-*O-H*-Phosphonoyl-3'-*O*-(1,1,3,3-tetra*iso*propyldisiloxan-1,3-diyl)-adenosine-2'-bis(4-(hydroxymethyl)phenylhexanoyl)-2'-phosphate (39). In a nitrogen atmosphere, 100 mg (1.50 mmol, 5.00 eq.) imidazole and 150 mL (1.05 mmol, 3.50 eq.) Et_3N were dissolved in dry CH_2Cl_2 and stirred at 0 °C for 15 minutes. After 28.8 µL (330 µmol, 1.10 eq.) PCl_3 were slowly added the reaction mixture was stirred for 15 minutes. The suspension was cooled to 5 °C and 300 mg (295 µmol, 1.00 eq.) Compound **38** dissolved in dry CH_2Cl_2 was added. The reaction solution was stirred for 15 minutes at room temperature. To terminate the reaction, a 1 M TEAB buffer solution was added and the mixture was stirred for 15 minutes at room temperature. The aqueous layer was extracted with CH_2Cl_2. The combined organic layers were dried over Na_2SO_4 and the solvent was removed in vacuo. The crude product was purified using automatic RP-18 chromatography (H_2O/CH_3CN gradient) and finally lyophilized. Yield: 312 mg (264 µmol, 89 %) as a colorless solid. The yield was calculated with one triethylammonium counterion. ^{1}H-NMR: (400 MHz, $CDCl_3$): *δ* 8.17 (s, 1H), 7.73 (s, 1H), 7.21-7.18 (m, 4H), 7.02-6.99 (m, 4H), 6.33 (bs, 2H), 6.15 (s, 1H), 5.05-5.02 (m, 1H), 4.93-4.85 (m, 6H), 4.35-4.16 (m, 2H), 3.05 (q, *J* = 7.4 Hz, 6H), 2.54 (t, *J* = 7.8 Hz, 4H), 1.78-1.71 (m, 4H), 1.43-1.37 (m, 8H), 1.30 (t, *J* = 7.3 Hz, 9H), 1.08-0.98 (m, 28H), 0.93 (t, *J* = 6.9 Hz, 6H); ^{13}C-NMR: (101 MHz, $CDCl_3$): *δ* 172.5, 156.8, 152.5, 150.4, 142.4, 137.3, 133.0, 129.6, 128.6, 122.4, 118.0, 93.3 (d, *J* = 9.1 Hz), 88.0 (d, *J* = 4.6 Hz), 78.2 (d, *J* = 5.5 Hz), 73.7 (d, *J* = 5.5 Hz), 70.0, 69.2, 64.1 (d, *J* = 5.5 Hz), 45.6, 34.5, 31.4, 24.7, 22.5, 17.6, 17.5, 17.3, 14.1, 13.8, 13.7, 13.6, 8.7; ^{31}P-NMR: (162 MHz, $CDCl_3$): *δ* 4.50, -1.53; IR: (ATR) $\tilde{\nu}$ [cm^{-1}]: 2921, 2853, 2378, 1683, 1612, 1513, 1062, 1004, 886, 519; HRMS (ESI, m/z): [M-H]$^-$ calcd. for $C_{48}H_{74}N_5O_{15}P_2Si_2$, 1078.4201; found, 1078.4028.

3'-*O*-(1,1,3,3-tetra*iso*propyldisiloxan-1,3-diyl)-adenosine-(2'-bis(4-(hydroxymethyl)phenylhexanoate)phosphoryl)-5'-phosphate (40). In a nitrogen atmosphere, 200 mg (169 µmol, 1.00 eq.) of compound **39** was dissolved in dry CH_2Cl_2 and 410 µL (1.69 mmol, 10.0 eq.) bis(trimethylsilyl)acetamide (BSA) was added. The solution was stirred for 1 h at room temperature. The solvent was removed under reduced pressure and the residue was dissolved in dry CH_2Cl_2 and 120 mg (507 µmol, 3.00 eq.) (1*S*)-(+)-(10-camphorsulfonyl)-oxaziridine (CSO) was added. After the reaction mixture was stirred for 1 h at room temperature the solvent was removed in vacuo. The residue was diluted in CH_3OH and an equal volume of 1 M TEAB buffer was added. After the reaction mixture was stirred at room temperature for 15 minutes it was concentrated to half the volume in vacuo. The aqueous layer was purified using automatic RP-18 chromatography (water/acetonitrile gradient) and finally lyophilized. Yield: 198 mg (165 µmol, 99 %) as a colorless solid. The yield was calculated with one triethylammonium counterion. ^{1}H-NMR: (400 MHz, CH_3OD): *δ* 8.68 (s, 1H), 8.17 (s, 1H), 7.37-7.30 (m, 2H), 7.15-7.12 (m, 4H), 7.02-6.99 (m, 4H), 6.41 (d, *J* = 7.0 Hz, 1H), 5.55-5.50 (m, 1H), 4.92-4.90 (m, 1H), 4.84-4.68 (m, 4H), 4.46-4.44 (m, 1H), 4.21-4.19 (m, 2H), 3.13 (q, *J* = 7.2 Hz, 6H), 2.57 (2xt *J* = 7.2 Hz, 4H), 1.77-1.70 (m, 4H), 1.44-

1.37 (m, 8H), 1.27 (t, *J* = 7.4 Hz, 9H), 1.16-1.03 (m, 28H), 0.95 (t, *J* = 6.9 Hz, 6H); ^{13}C-NMR: (101 MHz, CH_3OD): *δ* 173.6, 155.7, 152.5, 151.0, 141.8, 134.0, 130.5, 130.4, 130.3, 123.0, 122.9 (d, *J* = 4.8 Hz), 120.0, 87.3 (d, *J* = 9.2 Hz), 86.5 (d, *J* = 3.9 Hz), 80.3 (d, *J* = 5.1 Hz), 73.7 (d, *J* = 5.6 Hz), 70.6, 70.5 (d, *J* = 4.8 Hz), 65.8 (d, *J* = 5.1 Hz), 47.6, 35.0, 32.4, 25.6, 23.4, 18.1, 17.9, 17.8, 17.6, 14.9, 14.6, 14.3, 9.1; ^{31}P-NMR: (162 MHz, CH_3OD): *δ* 1.83, -1.02; IR: (ATR) $\tilde{\nu}$ [cm^{-1}]: 3292, 2942, 2865, 1755, 1691, 1514, 1462, 1246, 1200, 1080, 882, 828, 684; HRMS (ESI, m/z): [M-H]$^+$ calcd. for $C_{48}H_{74}N_5O_{16}P_2Si_2$, 1096.4295; found, 1096.4290. For Mass spectra (ESI$^+$) data see Supplementary Fig. 17.

3'-*O*-(1,1,3,3-Tetra*iso*propyldisiloxan-1,3-diyl)-MASTER-NADP (41). General procedure D with 59.9 µL (431 µmol, 10.0 eq.) TFAA, 95.5 µL (689 µmol, 16.0 eq.) Et_3N and 29.4 mg (43.1 µmol, 1.00 eq.) of the phosphate **32**, 20.6 µL (258 µmol, 6.00 eq.) NMI, 59.7 µL (431 µmol, 10.0 eq.) Et_3N and 42.0 mg (38.8 µmol, 0.90 eq.) of the phosphate **40** were used. The crude product was purified using automatic RP-18 chromatography (H_2O/CH_3CN gradient) and finally lyophilized. Yield: 51.3 mg (27.6 µmol, 64 %) as a colorless resin. The yield was calculated with two triethylammonium counterions. ^{1}H-NMR: (400 MHz, CH_3OD): *δ* 8.76 (s, 1H), 8.15 (s, 1H), 8.10 (s, 1H), 7.80 (d, *J* = 7.4 Hz, 1H), 7.63 (d, *J* = 7.8 Hz, 1H), 7.46-7.41 (m, 3H), 7.13-7.09 (m, 6H), 7.01-6.98 (m, 6H), 6.42 (d, *J* = 6.9 Hz, 1H), 5.60-5.55 (m, 1H), 4.96 (d, 1H, *J* = 5.5 Hz), 4.84-4.73 (m, 4H), 4.67-4.64 (m, 1H), 4.60 (s, 2H), 4.46-4.44 (m, 1H), 4.37-4.29 (m, 4H), 4.21 (t, *J* = 6.7 Hz, 2H), 4.20-4.19 (m, 2H), 4.06-4.03 (m, 1H), 3.15 (q, *J* = 6.7 Hz, 12H), 2.59 (t, *J* = 7.4 Hz, 4H), 1.80-1.69 (m, 6H), 1.47-1.34 (m, 16H), 1.27 (t, *J* = 7.3 Hz, 18H), 1.17-1.06 (m, 28H), 0.99-0.92 (m, 9H); ^{31}P-NMR: (162 MHz, CH_3OD): *δ* -2.42, -10.77 (d, *J* = 18.1 Hz), -11.42 (d, *J* = 22.4 Hz).

MASTER-NADP (42). A solution of 49.6 mg (26.6 µmol, 1.00 eq.) compound **41** in CH_3CN was cooled to 0 °C. To this mixture 1.92 µL (107 µmol, 4.00 eq.) water was added followed by 14.7 mg (53.3 µmol, 2.00 eq.) tris(dimethylamino)sulfonium difluorotrimethylsilicate (TASF). The reaction was stirred for 1 h at 0 °C and terminated with silica gel. The suspension was filtered, and the solvent was removed in vacuo. The crude product was purified using automatic RP-18 chromatography (H_2O/CH_3CN gradient) and finally lyophilized. Yield: 23.5 mg (14.6 µmol, 55 %) as a colorless solid. The yield was calculated with two triethylammonium counterions. ^{1}H-NMR: (500 MHz, CH_3OD): *δ* 8.63 (s, 1H), 8.14 (s, 1H), 8.02 (s, 1H), 7.72 (d, *J* = 7.6 Hz, 1H), 7.55 (d, *J* = 7.2 Hz, 1H), 7.38-7.34 (m, 3H), 7.06-7.04 (m, 6H), 6.90-6.88 (m, 4H), 6.11 (d, *J* = 6.9 Hz, 1H), 5.17-5.16 (m, 1H), 4.94 (d, *J* = 6.2 Hz, 1H), 4.72-4.70 (m, 4H), 4.60-4.58 (m, 1H), 4.53 (s, 2H), 4.43-4.40 (m, 1H), 4.27-4.20 (m, 4H), 4.15 (t, *J* = 6.7 Hz, 2H), 4.12-4.06 (m, 2H), 3.98-3.96 (m, 1H), 3.08 (q *J* = 7.2 Hz, 12H,), 2.50 (t, *J* = 7.2 Hz, 4H), 1.69-1.63 (m, 6H), 1.36-1.24 (m, 16H), 1.21 (t, *J* = 7.4 Hz, 18H), 0.91-0.84 (m, 9H); ^{31}P-NMR: (162 MHz, CH_3OD): *δ* -2.42, -10.80 (d, *J* = 17.8 Hz), -11.42 (d, *J* = 21.8 Hz).

Supplementary Fig. 14: Mass spectra (ESI+) of 2'-(Bis-*O*-(4-heptanoyloxybenzyl))-(3'-deoxy-3'-fluoro-adenosine)-2',5'-diphosphate (15)

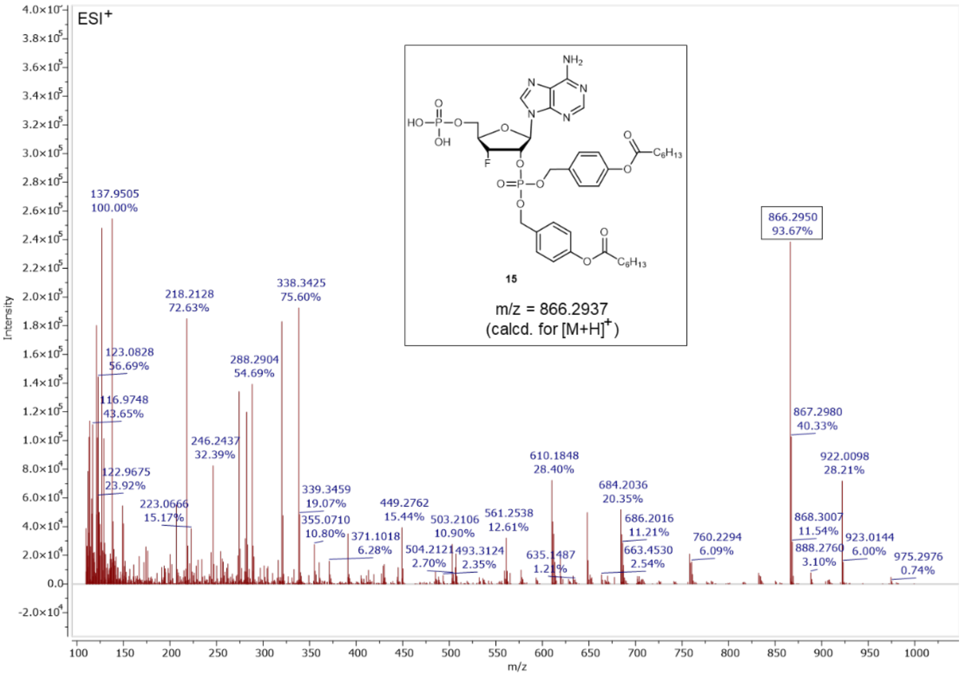

Mass spectra (ESI+) of 2'-(Bis-*O*-(4-heptanoyloxybenzyl))-(3'-deoxy-3'-fluoro-adenosine)-2',5'-diphosphate (**15**). The sample was dissolved in dichloromethane and diluted with acetonitrile (1 µg/mL). 5 µL were injected and measured with electron spray ionization time of flight mass spectrometry (Agilent 6224 ESI-TOF instrument). The measurement was done in positive mode with a mass range of m/z 110-3200 and a rate of 1.03 spectra/s. The gas temperature was set to 325 °C and the drying gas flow to 10 L/min.

Supplementary Fig. 15: Mass spectra (ESI$^-$) of ((Ribofuranosyl)-1-(4-(((heptyloxy)carbonyl)oxy)benzyl-benzoyl)-5-phosphate (25)

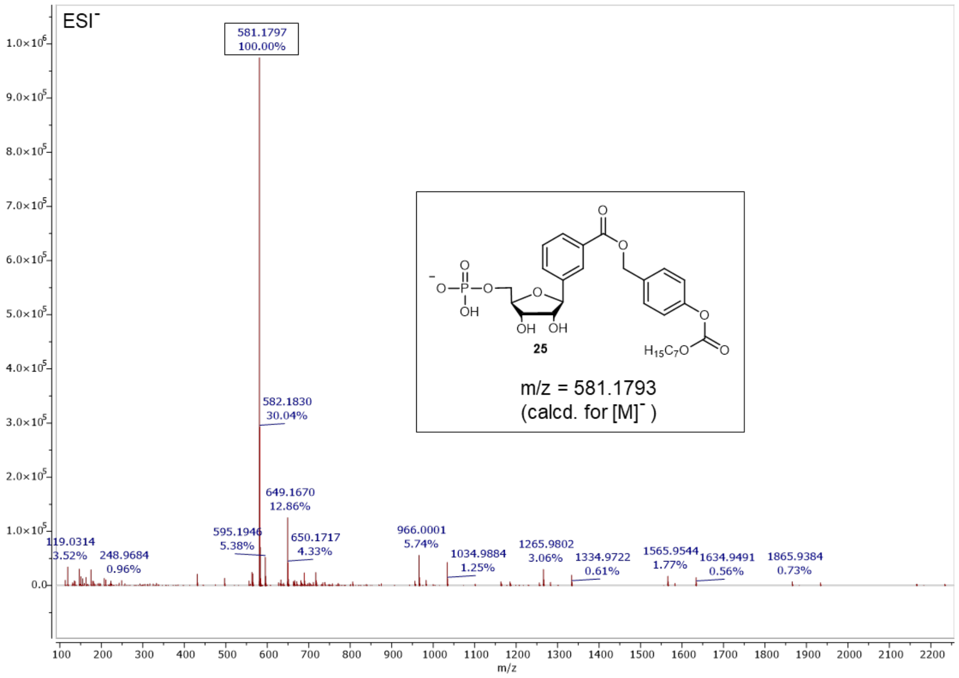

Mass spectra (ESI$^-$) of ((Ribofuranosyl)-1-(4-(((heptyloxy)carbonyl)oxy)benzyl-benzoyl)-5-phosphate (**25**). The sample was dissolved in dichloromethane and diluted with acetonitrile (10 µg/mL). 0.5 µL were injected and measured with electron spray ionization time of flight mass spectrometry (Agilent 6224 ESI-TOF instrument). The measurement was done in negative mode with a mass range of m/z 110-3200 and a rate of 1.03 spectra/s. The gas temperature was set to 325 °C and the drying gas flow to 10 L/min.

Supplementary Fig. 16: Mass spectra (ESI$^-$) of MASTER-NAADP (26)

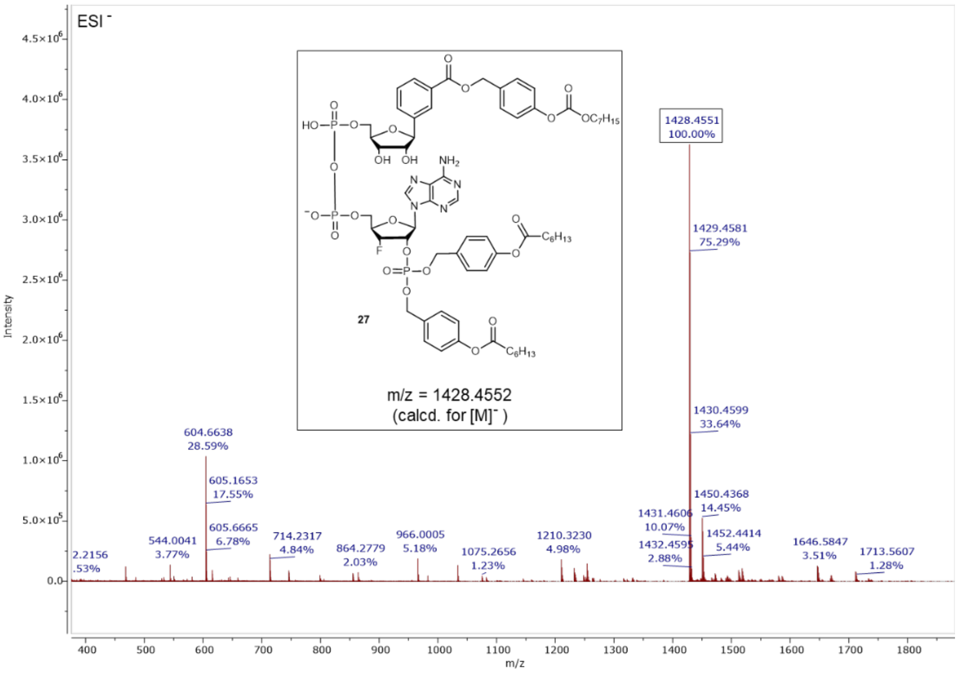

Mass spectra (ESI$^-$) of MASTER-NAADP (**26**). The sample was dissolved in water and diluted with acetonitrile (10 µg/mL). 2.5 µL were injected and measured with electron spray ionization time of flight mass spectrometry (Agilent 6224 ESI-TOF instrument). The measurement was done in negative mode with a mass range of m/z 110-3200 and a rate of 1.03 spectra/s. The gas temperature was set to 325 °C and the drying gas flow to 10 L/min.

Supplementary Fig. 17: Mass spectra (ESI+) of 3'-*O*-(1,1,3,3-tetra*iso*propyldisiloxan-1,3-diyl)-adenosine-(2'-bis(4-(hydroxymethyl)phenylhexanoate)phosphoryl)-5'-phosphate (40)

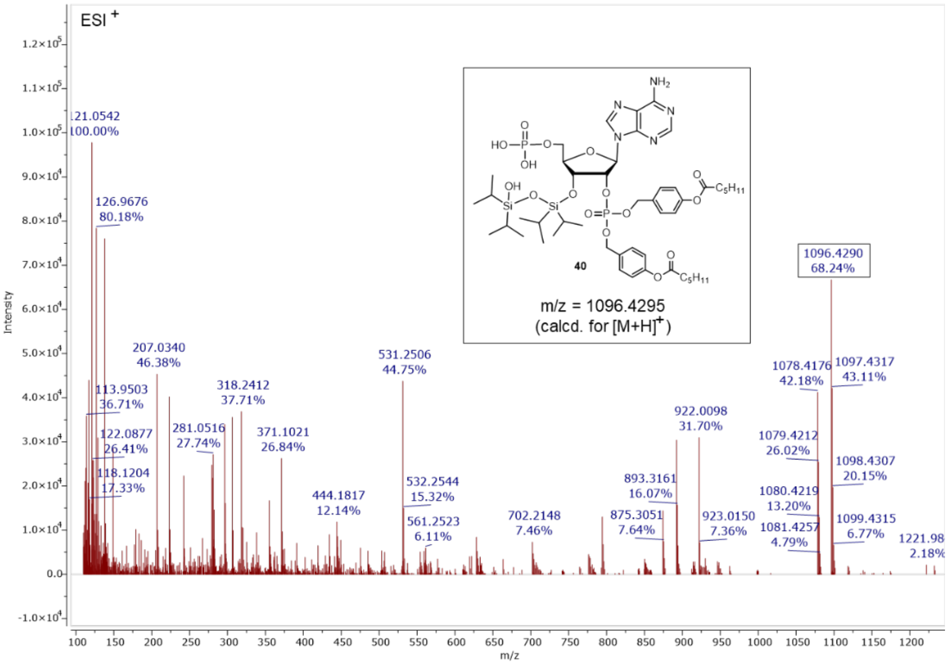

Mass spectra (ESI+) of 3'-*O*-(1,1,3,3-tetra*iso*propyldisiloxan-1,3-diyl)-adenosine-(2'-bis(4-(hydroxy-methyl)phenylhexanoate)phosphoryl)-5'-phosphate (**40**). The sample was dissolved in acetonitrile and diluted with acetonitrile (10 µg/mL). 0.5 µL were injected and measured with electron spray ionization time of flight mass spectrometry (Agilent 6224 ESI-TOF instrument). The measurement was done in positive mode with a mass range of m/z 110-3200 and a rate of 1.03 spectra/s. The gas temperature was set to 325 °C and the drying gas flow to 10 L/min.
