# Supplementary material for: MASTER-NAADP: a membrane permeable precursor of the Ca2+ mobilizing second messenger NAADP
Source: Nat Commun. 2024 Sep 13;15:8008. doi: 10.1038/s41467-024-52024-y (PMC11399135; doi:10.1038/s41467-024-52024-y)
Supplement: Supplementary file 3 — Reporting Summary [file 41467_2024_52024_MOESM3_ESM.pdf]

nature portfolio

Corresponding author(s): Andreas H Guse

Last updated by author(s): Aug 19, 2024

Reporting Summary

Nature Portfolio wishes to improve the reproducibility of the work that we publish. This form provides structure for consistency and transparency in reporting. For further information on Nature Portfolio policies, see our Editorial Policies and the Editorial Policy Checklist.

Statistics

For all statistical analyses, confirm that the following items are present in the figure legend, table legend, main text, or Methods section.

n/a	Confirmed	
☐	☒	The exact sample size (*n*) for each experimental group/condition, given as a discrete number and unit of measurement
☐	☒	A statement on whether measurements were taken from distinct samples or whether the same sample was measured repeatedly
☐	☒	The statistical test(s) used AND whether they are one- or two-sided<br>*Only common tests should be described solely by name; describe more complex techniques in the Methods section.*
☒	☐	A description of all covariates tested
☐	☒	A description of any assumptions or corrections, such as tests of normality and adjustment for multiple comparisons
☐	☒	A full description of the statistical parameters including central tendency (e.g. means) or other basic estimates (e.g. regression coefficient) AND variation (e.g. standard deviation) or associated estimates of uncertainty (e.g. confidence intervals)
☐	☒	For null hypothesis testing, the test statistic (e.g. *F*, *t*, *r*) with confidence intervals, effect sizes, degrees of freedom and *P* value noted<br>*Give P values as exact values whenever suitable.*
☒	☐	For Bayesian analysis, information on the choice of priors and Markov chain Monte Carlo settings
☒	☐	For hierarchical and complex designs, identification of the appropriate level for tests and full reporting of outcomes
☒	☐	Estimates of effect sizes (e.g. Cohen's *d*, Pearson's *r*), indicating how they were calculated

Our web collection on statistics for biologists contains articles on many of the points above.

Software and code

Policy information about availability of computer code

Data collection	HPLC: OpenLAB CDS ChemStation Edition C.01.05 (Agilent); MassSpec: MestreNova software (Mestrelab Research); Imaging: Volocity software (version 6.6.2; PerkinElmer Inc.); Molecular Modeling: MAESTRO (version 12.6; Schrödinger); Western blot: EVOLUTION-CAPT (Vilber); Fluorimeter: FL-Solution (version 4.2; Hitachi) + Hitachi Calcium Application Software; Lipid planar bilayer: Clampfit (version 10.1; Molecular Devices)
Data analysis	Sequence analysis with the aid of the designated algorithm "Interference of CRISPR Edits" (ICE). Statistic: Prism10 (graphpad) and Excel (16.86; Microsoft); Global Ca2+ imaging: FIJI (ImageJ; Version 2.0.0) . Image processing and analysis (Ca2+ microdomain analysis) as described in Diercks et al., Methods Mol Biol 2019:1929. The image data analysis software is available upon request. A Python implementation of the Ca2+ image shape-normalisation and dartboard projections analysis routines is publicly available (license: Apache 2.0) at Kovacevic D, Woelk L-M, Husseini H, Förster F, Werner R. IPMI-ICNS-UKE/DARTS: DARTS FIMMU 2024 release (Version 1.0.0). Zenodo. https://zenodo.org/doi/10.5281/zenodo.10459242. The corresponding documentation can be found in Woelk, L.-M. et al. DARTS: an open-source Python pipeline for Ca2+ microdomain analysis in live cell imaging data. Frontiers in Immunology 14, (2024).

For manuscripts utilizing custom algorithms or software that are central to the research but not yet described in published literature, software must be made available to editors and reviewers. We strongly encourage code deposition in a community repository (e.g. GitHub). See the Nature Portfolio guidelines for submitting code & software for further information.

Data

Policy information about availability of data

All manuscripts must include a data availability statement. This statement should provide the following information, where applicable:

- Accession codes, unique identifiers, or web links for publicly available datasets
- A description of any restrictions on data availability
- For clinical datasets or third party data, please ensure that the statement adheres to our policy

A material transfer agreement between the University Medical Center Hamburg-Eppendorf, Hamburg, Germany, and potentially interested academic recipients exists for material produced by The Calcium Signaling Group, Department of Biochemistry and Molecular Cell Biology, University Medical Center Hamburg-Eppendorf and/or Organic Chemistry, University of Hamburg.
The authors declare that the data supporting the findings of this study are available within the paper and its Supplementary Information files. Large high-resolution imaging raw data files that were generated and used in this study are available from the corresponding author upon request. Source data are provided with this paper.

Research involving human participants, their data, or biological material

Policy information about studies with human participants or human data. See also policy information about sex, gender (identity/presentation), and sexual orientation and race, ethnicity and racism.

Reporting on sex and gender	*Use the terms sex (biological attribute) and gender (shaped by social and cultural circumstances) carefully in order to avoid confusing both terms. Indicate if findings apply to only one sex or gender; describe whether sex and gender were considered in study design; whether sex and/or gender was determined based on self-reporting or assigned and methods used.<br>Provide in the source data disaggregated sex and gender data, where this information has been collected, and if consent has been obtained for sharing of individual-level data; provide overall numbers in this Reporting Summary. Please state if this information has not been collected.<br>Report sex- and gender-based analyses where performed, justify reasons for lack of sex- and gender-based analysis.*
Reporting on race, ethnicity, or other socially relevant groupings	*Please specify the socially constructed or socially relevant categorization variable(s) used in your manuscript and explain why they were used. Please note that such variables should not be used as proxies for other socially constructed/relevant variables (for example, race or ethnicity should not be used as a proxy for socioeconomic status).<br>Provide clear definitions of the relevant terms used, how they were provided (by the participants/respondents, the researchers, or third parties), and the method(s) used to classify people into the different categories (e.g. self-report, census or administrative data, social media data, etc.)<br>Please provide details about how you controlled for confounding variables in your analyses.*
Population characteristics	*Describe the covariate-relevant population characteristics of the human research participants (e.g. age, genotypic information, past and current diagnosis and treatment categories). If you filled out the behavioural & social sciences study design questions and have nothing to add here, write "See above."*
Recruitment	*Describe how participants were recruited. Outline any potential self-selection bias or other biases that may be present and how these are likely to impact results.*
Ethics oversight	*Identify the organization(s) that approved the study protocol.*

Note that full information on the approval of the study protocol must also be provided in the manuscript.

Field-specific reporting

Please select the one below that is the best fit for your research. If you are not sure, read the appropriate sections before making your selection.

☒ Life sciences ☐ Behavioural & social sciences ☐ Ecological, evolutionary & environmental sciences

For a reference copy of the document with all sections, see nature.com/documents/nr-reporting-summary-flat.pdf

Life sciences study design

All studies must disclose on these points even when the disclosure is negative.

Sample size	No sample-size calculation was performed. However, from previous publications (Gu et al. 2021 PMID: 34784249, Roggenkamp et al. 2021 PMID: 33758062, Diercks et al. 2018 PMID: 30563862, Brock et al. 2022 PMID: 35119925) we know for initial calcium microdomains that at least 20 cells from 3 individual experiments are needed to obtain robust results in T cells. For global calcium measurements in T cells approximately 80 - 100 cells per condition from at least 3 individual experiments are sufficient (Gu et al. 2021 PMID: 34784249, Roggenkamp et al. 2021 PMID: 33758062, Brock et al. 2022 PMID: 35119925). Similar sample sizes were applied to Neuro2a cells and KHYG-1 cells.
Data exclusions	Cells were excluded if they had a cytosolic calcium concentration above baseline before stimulation, or cells that did not respond to a positive control, e.g. by addition of SERCA inhibitor thapsigargin.

Replication	At least three independent replicates were performed for all experiments.
Randomization	Randomized data acquisition was performed for each imaging day and conditions.
Blinding	This is not applicable to our study design.

Reporting for specific materials, systems and methods

We require information from authors about some types of materials, experimental systems and methods used in many studies. Here, indicate whether each material, system or method listed is relevant to your study. If you are not sure if a list item applies to your research, read the appropriate section before selecting a response.

Materials & experimental systems

n/a	Involved in the study	
☐	☒	Antibodies
☐	☒	Eukaryotic cell lines
☒	☐	Palaeontology and archaeology
☐	☒	Animals and other organisms
☒	☐	Clinical data
☒	☐	Dual use research of concern
☒	☐	Plants

Methods

n/a	Involved in the study	
☒	☐	ChIP-seq
☒	☐	Flow cytometry
☒	☐	MRI-based neuroimaging

Antibodies

Antibodies used	OKT3 (human monoclonal anti-CD3 antibody) self manufactured; polyclonal HN1L antibody (dilution: 1:1000; Lot-ID: 1625; catalog ID: orb1412; manufacturer: Biorbyt); polyclonal HN1L antibody (dilution: 1:2000; catalog ID: HPA041888; manufacturer: Atlas Antibodies); anti-rabbit secondary antibody conjugated with horseradish peroxidase (dilution: 1:5000; catalog ID: ab6721; manufacturer: Abcam); peroxidase AffiniPure goat anti-rabbit immunoglobulin G (dilution: 1:20.00; catalog ID: 111-035-045; manufacturer: Jackson ImmunoResearch Europe LTD.); anti-α-actin (dilution: 1:500; catalog ID: MAB1501; manufacturer: Merck) or anti-β-actin (dilution: 1:500; catalog ID: SC-47778; manufacturer: Santa Cruz);
Validation	HN1L antibody (Biorbyt): tested applications by manufacturer: ELISA, IF, IHC-P, IHC-Fr; HN1L antibody (Atlas Antibodies): tested applications by manufacturer: ICC-IF, IHC, WB; anti-α-actin (Merck): tested by manufacturer: ELSIA, ICC, IF, IHC (p), WB; anti-β-actin (Santa Cruz): tested by manufacturer: WB, IP, IF, IHC(P), ELISA; anti-rabbit secondary antibody conjugated with horseradish peroxidase (Abcam): tested by manufacturer: Dot, IM, ELISA, ICC, IHC-Fr, IHC-P, WB; peroxidase AffiniPure goat anti-rabbit immunoglobulin G (ackson ImmunoResearch Europe LTD.): tested by manufacturer: WB, ELISA, IHC;<br>OKT3: Lau & Goldstein 1981 (PMID: 6457004) based on this publication from the described OKT3 hybridoma cell OKT3 was self manufactured and used in several publications (Wolf et al. 2015, Diercks et al. 2018, Roggenkamp et al. 2021)

Eukaryotic cell lines

Policy information about cell lines and Sex and Gender in Research

Cell line source(s)	Neuro2a (N2a) cells were obtained from the DSMZ German Collection of Microorganisms and Cell Cultures GmbH.<br>KHYG-1 were kindly gifted by Prof. Marcus Altfeld, Leibnitz Institute for Virology, Hamburg.<br>Jurkat subclone JMP was originally generated by the University of Erlangen Medical Faculty (Germany).
Authentication	N2a cell line was directly obtained from DSMZ and is thereby authenticated.<br>KHYG-1 were directly obtained from DSMZ in April 2016 by Prof. Marcus Altfeld and were thereby authenticated.<br>Jurkat subclone JMP were authenticated as Jurkat cells by short tandem repeat profiling and tested negative for contamination with rodent cells (DSMZ service for authentication of human cell lines) (Fliegert et al. 2017).
Mycoplasma contamination	All cell cultures are tested every quaterly for Mycoplasma in our facility
Commonly misidentified lines<br>(See ICLAC register)	not applicable

Animals and other research organisms

Policy information about studies involving animals; ARRIVE guidelines recommended for reporting animal research, and Sex and Gender in Research

Laboratory animals	Spleen and lymph nodes of WT mice (C57BL/6J; age 6-12 weeks; Mus musculus) were required for studies on primary murine CD4+ T cells. Housing was performed under standardized conditions at a 12/12-h light-dark cycle with food and water supply ad libitum in the animal facility of the University Medical Center Hamburg- Eppendorf (UKE).

Wild animals	not applicable
Reporting on sex	Sex was not considered in this study design. Both female and male mice were included in our study.
Field-collected samples	not applicable
Ethics oversight	All mice experiments were approved by the Animal Welfare Officers of UKE and Behörde für Gesundheit und Verbraucherschutz Hamburg (ORG934).

Note that full information on the approval of the study protocol must also be provided in the manuscript.
